# Suppression of mPFC‐Amygdala Circuit Mitigates Sevoflurane‐Induced Cognitive Deficits in Aged Mice

**DOI:** 10.1111/cns.70443

**Published:** 2025-05-16

**Authors:** Junhua Li, Jinbei Wen, Meigu Zeng, Jinghong Mei, Cong Zeng, Ning Liufu, Yujuan Li

**Affiliations:** ^1^ Department of Anesthesiology, Sun Yat‐sen Memorial Hospital Sun Yat‐sen University Guangzhou China; ^2^ Guangdong Provincial Key Laboratory of Malignant Tumor Epigenetics and Gene Regulation, Sun Yat‐sen Memorial Hospital Sun Yat‐sen University Guangzhou China; ^3^ Medical Research Center of Shenshan Medical Center Sun Yat‐sen Memorial Hospital Shanwei China; ^4^ Guangdong Province Key Laboratory of Brain Function and Disease, Zhongshan School of Medicine Sun Yat‐sen University Guangzhou China; ^5^ Brain Research Center, Sun Yat‐sen Memorial Hospital Sun Yat‐sen University Guangzhou China

**Keywords:** inflammatory response, mitochondrial stress, neuronal circuit, perioperative neurocognitive disorders, sevoflurane

## Abstract

**Background:**

Perioperative neurocognitive disorders (PND) are common and costly complications in elderly surgical patients, yet the involvement of specific neural circuits in their etiology remains poorly understood. We hypothesized that neural projections from the medial prefrontal cortex (mPFC) to the amygdala contribute to PND pathogenesis.

**Methods:**

Using chemogenetic approaches, we selectively suppressed or excited the mPFC and its projections to the amygdala in a murine model exposed to sevoflurane. We assessed cognitive deficits, synaptic plasticity (AMPA receptor activity, long‐term potentiation [LTP]), mitochondrial stress, neuroinflammatory markers, and neuronal apoptosis in the amygdala. Additional interventions included pharmacological suppression of AMPA receptors, glutamate biosynthesis, and mitochondrial stress within the amygdala.

**Results:**

Sevoflurane exposure activated the mPFC‐amygdala circuit. Chemogenetic suppression of the mPFC attenuated sevoflurane‐induced cognitive deficits, AMPA receptor hyperexcitation, mitochondrial dysfunction, neuroinflammation, and neuronal apoptosis in the amygdala. Retrograde inhibition of mPFC projections to the amygdala alleviated cognitive impairments, whereas retrograde excitation exacerbated them. Suppressing AMPA receptors, glutamate synthesis, or mitochondrial stress in the amygdala similarly reduced cognitive deficits and pathological alterations. Notably, mPFC suppression rescued sevoflurane‐induced LTP impairment in the amygdala.

**Conclusions:**

These findings demonstrate that sevoflurane activates the mPFC‐amygdala circuit, driving PND‐associated cognitive deficits and neuropathological changes. Targeting this circuit or downstream mechanisms (AMPA signaling, mitochondrial stress) may mitigate sevoflurane‐induced PND. This study provides empirical evidence implicating specific neural circuitry in anesthetic‐related neurocognitive dysfunction.

## Graphical abstract

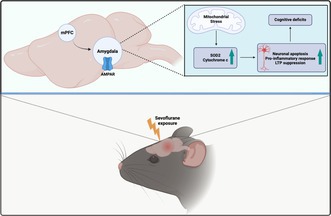

## 1 Introduction

Up to 40% of aged patients experience neurological complications, known as perioperative neurocognitive disorders (PNDs). PNDs are common complications in elder patients after surgery and anesthesia, marked by an impairment in learning and memory functions [1, 2]. This condition may persist for several months, severely diminishing the patient's quality of life and imposing considerable strain on familial and societal resources [3]. Sevoflurane, a volatile anesthetic, may contribute to PNDs, particularly in elderly patients. It can cause neuroinflammation and neuronal damage, affecting synaptic plasticity and cognition [4, 5, 6]. Prolonged or repeated exposure to sevoflurane raises the risk of PND, especially in those with pre‐existing cognitive dysfunction [7, 8]. Consequently, there is a pressing imperative to delineate efficacious strategies to ameliorate PND. Addressing this clinical challenge necessitates a deeper comprehension of the underlying pathophysiological mechanisms that contribute to the development of PND. PND may manifest as disturbances across a spectrum of cognitive domains, encompassing long‐term memory retention, cognitive adaptability, working memory, focused attention, language comprehension, and cognitive alacrity [9]. While the medial prefrontal cortex (mPFC) helps regulate the amygdala's response to stress and emotion, the involvement of dysfunction in this circuitry in PND remains underexplored.

In rodents, mPFC encompasses the infralimbic cortex, anterior cingulate cortex, and prelimbic cortex, with each subdivision exhibiting unique patterns of connectivity and functional attributes [10, 11]. Collectively, these regions exhibit dense interconnectivity with other cortical association areas, midline thalamic nuclei, the limbic system, and a diverse collection of midbrain and brainstem nuclei with distinct behavioral functionalities [12, 13]. Via this array of inputs and outputs, the mPFC assumes a critical role in orchestrating decision‐making processes, memory consolidation, social interactions, mood regulation, and cognitive functions [14, 15]. mPFC collaborates with the dorsal hippocampus in the facilitation of spatial information processing, a function reliant on the hippocampus [16]. Actually, mPFC and hippocampus are recognized for their pivotal contributions to cognition, emotional regulation, and sensory processing [17, 18, 19]. The mPFC is implicated in executive function and memory consolidation, and preclinical work links its dysregulation to neurocognitive deficits [20]. While the hippocampus is often studied in PND, the mPFC's connectivity with limbic regions like the amygdala—central to emotional memory and stress responses—positions it as a key mediator of anesthesia‐related cognitive decline [21]. Given its diverse cognitive functions and strong connections with the hippocampus, the mPFC is likely involved in the etiology of PND.

The amygdala has been historically recognized for its involvement in cognition and emotion [22, 23]. Nonetheless, the role of the amygdala in mediating PND remains unclear, and the neural pathways underlying PND have yet to be delineated. These pathways involve projections from the mPFC, primarily consisting of glutamatergic projection neurons, along with a smaller proportion of GABAergic interneurons [24, 25]. In the amygdala, axonal projections from mPFC glutamatergic neurons represent the primary source of synaptic connections [26]. The mPFC‐amygdala neural circuit has been implicated in regulating cognitive and emotional processes [27, 28]. Consequently, we hypothesize that sevoflurane exposure may influence cognitive functions through modifications in the mPFC‐amygdala circuit. Viral tracing strategies, animal behavioral experiments, immunoblotting, immunofluorescence, and chemogenetic methodologies were employed within a mouse model of PND to verify this hypothesis. The influence of the mPFC‐amygdala circuit on PND was assessed by inhibiting neurons within the mPFC or amygdala. In this study, we demonstrated that the mPFC‐amygdala neuronal circuit was activated by sevoflurane exposure. The stimulation of projections from the mPFC to the amygdala induced mitochondrial stress within the amygdala. Chemogenetic suppression of the mPFC‐amygdala circuit mitigated sevoflurane‐induced cognitive impairments. These findings highlight the role of the mPFC‐amygdala circuit in the pathogenesis of sevoflurane‐induced PND.

## 2 Methods and Materials

### 2.1 Animal

Aged C57BL/6 mice (18 months old, male) were used in the present study. Since investigating sex differences was not the primary focus of our study, female mice were excluded from the experiments. Mice were housed on a 12‐h light/dark cycle (lights on at 7:00 AM), and testing during the light phase aligns with their inactive period, ensuring uniform baseline activity across experimental groups. All behavioral tests were conducted during the light phase (9:00 AM to 5:00 PM) under standardized lighting conditions to minimize circadian variability, consistent with standard protocols for rodent behavioral assessments. This study was conducted in accordance with the guidelines for animal care set by the National Institutes of Health (NIH). The study protocols were approved by the Animal Care Committee of Sun Yat‐sen University, Guangzhou, China.

### 2.2 Sevoflurane Exposure

As we previously described, the mice were anesthetized through inhalation of a gas mixture consisting of 3% sevoflurane and 60% oxygen [29]. The animals were placed in a chamber for 4 h, with the concentrations of oxygen and sevoflurane continuously monitored using a gas monitor (Mindray, China). A heating pad was used to maintain a stable body temperature of 36.5°C ± 0.5°C, with rectal temperature monitored continuously during the procedure. The Con group underwent the same procedural steps but was not exposed to sevoflurane.

### 2.3 Viral and Chemical Injections

Viral vectors, such as AAV2‐hSyn‐mCherry, AAV2‐hSyn‐hM4Di‐mCherry, AAV2/9‐hSyn‐DIO‐hM4Di‐mCherry, AAV2/9‐hSyn‐DIO‐mCherry, and AAV2/9‐hSyn‐DIO‐hM3Dq‐mCherry (Hanbio, China), were stereotaxically administered bilaterally into the mPFC (1.8 mm anterior to bregma, ± 0.4 mm from the midline, and 1.8 mm below the skull surface). A volume of 200 nL per side was injected by employing a 1 μL Hamilton Neuros syringe (25 nL/min). In a similar manner, AAV2/2 retro plus‐hSyn‐Cre‐WPRE‐pA (Genechem, China) was stereotaxically injected bilaterally into the amygdala at coordinates: −1.2 mm to −1.7 mm posterior to bregma, ±2.8 mm to ±3.2 mm from the midline, and −4.5 mm to −5.2 mm from the skull surface. A total volume of 300 nL per side was injected (50 nL/min). After the viral injections, the mice were placed back in their cages and given a recovery period of at least 3 weeks before being subjected to behavioral tests or other experiments.

Additionally, pharmacological agents were administered to explore their effects on the experimental model. The AMPA receptor antagonist CNQX (300 μg/mL) (Sigma‐Aldrich), the glutaminase inhibitor BSO (100 μg/mL) (Sigma‐Aldrich), or the mitochondrial stress antagonist NAC (200 μg/mL) (Cayman Chemical) were stereotaxically injected bilaterally into the amygdala (0.4 μL per side, 1 nL/s) employing a micro syringe pump controller (NanoJect III, Drummond Scientific Company). After 30 min, the mice were exposed to sevoflurane. Lastly, clozapine‐N‐oxide (CNO) (Tocris), dissolved in dimethyl sulfoxide (DMSO) (Sigma‐Aldrich) to a concentration of 2 mg/mL, was injected intraperitoneally. At least 3 weeks after the injection of hM4Di or hM3Dq viral vectors, the mice were given CNO or an equivalent volume of DMSO intraperitoneally, 1 h before sevoflurane exposure.

### 2.4 Open Field Test

As previously described, the open field test was conducted on the 3rd day following sevoflurane exposure [30]. In brief, the mice were placed in the open field arena for a 5‐min period. Video recordings were made of the mice's behaviors during the test, and EthoVision XT 7.0 software (Noldus, Netherlands) was used to analyze the traveling speed, distance traveled, and duration in the center. The box was thoroughly disinfected using 75% alcohol to eliminate any potential odors from animal waste, thereby minimizing distractions before introducing the next mouse.

### 2.5 Novel Object Recognition (NOR) Test

On the 4th day following sevoflurane exposure, the NOR test was conducted. During the adaptation phase, the mice were placed in the box with two identical objects and given 5 min to explore freely. One of the objects was substituted with a novel object 4 h after the training trial. The mice were then given 5 min to explore. The frequency of interactions and the duration spent with each object were recorded. The new object recognition index was calculated as the ratio of the number of times the mice explored the novel object to the total number of object explorations. Additionally, the exploration rate was defined as the time spent exploring the novel object relative to the total time spent exploring both objects.

### 2.6 Morris Water Maze (MWM)

On the 5th day following sevoflurane exposure, the MWM test was performed to evaluate the spatial learning and memory capabilities of aged mice [29]. Prior to the testing phase, the mice underwent a 2‐day acclimatization period in the water maze apparatus. The mice were placed in one of three distinct quadrants and trained to find a submerged platform over 5 consecutive days during the training phase. If the animal was unable to locate the platform within 1 min, it was gently directed to the platform and allowed to stay there for 10 s. A probe trial was conducted 24 h after the final training session to evaluate memory retention, during which the platform was removed. Memory performance was quantified by recording the time spent in the target quadrant and platform crossing times.

### 2.7 Fear Conditioning Test

On the 11th day after sevoflurane exposure, the fear conditioning test was conducted to evaluate the associative memory of the mice [31]. The aged mice were placed in a conditioning chamber (Panlab, Barcelona), equipped with a ventilation fan to generate background noise. Following the conditioning stimulus, the mouse was removed from the chamber 30 s after the final pairing. The animal was placed back in the same chamber 24 h later for a 6‐min session but without exposure to the tone or shock. Two hours after this, the mouse was placed in a different chamber with a distinct context and scent. Freezing activity was observed for 3 min without the tone. Subsequently, the tone was delivered for 3 cycles, each lasting 30 s, with a 1‐min pause between cycles. The freezing duration of aged mice during context and tone‐associated fear conditioning tests was measured.

### 2.8 Western Blot

The mPFC and amygdala were homogenized in lysis buffer with RIPA (Beyotime, China) and PMSF (Beyotime, China) in a 100:1 ratio for protein extraction. The samples were loaded onto SDS‐PAGE gels for separation by molecular weight, followed by transfer to nitrocellulose membranes. After blocking the membrane with 5% nonfat milk in TBST, it was incubated overnight at 4°C with the following primary antibodies: rabbit anti‐p‐GluA1 (Cell signaling technology, Cat #75574, 1:1000), rabbit anti‐GluA1 (Cell signaling technology, Cat#13185, 1:1000), rabbit anti‐SOD2 (Cell signaling technology, Cat#13141, 1:1000), rabbit anti‐cytochrome c (Cell signaling technology, Cat#11940, 1:1000), rabbit anti‐cleaved caspase‐3 (Cell signaling technology, Cat#9664, 1:1000), rabbit anti‐caspase‐3 (Cell signaling technology, Cat #14220, 1:1000), and mouse anti‐β‐actin (Abcam, Cat#ab6276, 1:1000). After washing with TBST three times, the membranes were incubated with HRP‐conjugated secondary antibodies for detection. Enhanced chemiluminescence was employed to visualize the protein bands, and protein quantification was performed using ImageJ software (National Institutes of Health, USA).

### 2.9 In Vitro Electrophysiology

In vitro electrophysiological ACSF was prepared using the following protocol: 25 mM NaHCO_3_, 2 mM CaCl_2_, 2.5 mM KCl, 1 mM MgCl_2_, 124 mM NaCl, 10 mM D‐glucose, and 1 mM NaH_2_PO_4_, adjusted to pH 7.35–7.45. Prior to use, ACSF was balanced with a gas mixture consisting of 95% O_2_ and 5% CO_2_ (v/v). Coronal sections (300 μm thick) of the amygdala, the designated brain area, were prepared with a vibratome in oxygenated ice‐cold ACSF. The brain slices were incubated in oxygenated ACSF at 30°C ± 1°C for 1.5 h to promote recovery. After the recovery phase, a single slice was positioned on the microelectrode array system probe (MED‐PG515A) located on the microscope stage. Once the slice stabilized, a fine mesh and anchor were carefully positioned on top to maintain stability during recording. During electrophysiological recordings, the slices were continuously supplied with oxygenated ACSF at 30°C ± 1°C using a peristaltic pump. To determine the best stimulation location, monopolar, biphasic constant‐current pulses (0.2 ms duration) were administered via Mobius software, with a 2‐s interval between each pulse. After 30 min of recovery, during which baseline fEPSP responses stabilized, an input–output curve was generated by measuring the fEPSP amplitude in response to a series of escalating stimulation intensities in 10 μA increments. To induce LTP, the baseline synaptic response was defined by selecting a stimulation intensity that corresponded to 30%–50% of the saturation point. Stable fEPSPs were recorded for at least 15 min as the baseline. The fEPSP amplitude changes were assessed as a percentage relative to the baseline. To compare LTP magnitude between groups, the average values from the final 10 min were subjected to statistical analysis. Slices exhibiting unstable baselines were excluded from the analysis.

### 2.10 Immunofluorescent Staining

Immunofluorescence staining was performed as outlined in our prior study [29]. In brief, samples were gathered and immersed in 4% paraformaldehyde for a 24‐h period. Coronal sections of the brain, measuring between 50 and 60 μm, were prepared utilizing a vibratome (Leica, Germany). The tissue samples underwent a permeabilization process by being incubated in a 0.2% Triton X‐100 solution for 1 h at around 24°C. This was subsequently followed by a blocking step where the samples were treated with a mixture containing 0.05% Triton X‐100 and 10% bovine serum albumin for an hour at room temperature. The tissues were subsequently rinsed once with phosphate‐buffered saline (PBS). Following this, they were incubated for 24 h at 4°C with primary antibodies that had been diluted in PBS with 0.2% Triton X‐100. The primary antibodies used in this investigation consisted of rabbit anti‐c‐Fos (Cell Signaling Technology, Cat#2250, dilution 1:500), mouse anti‐NeuN (Abcam, Cat#ab104224, dilution 1:500), rabbit anti‐VGAT (Abcam, Cat#ab307448, dilution 1:500), and rabbit anti‐VGLUT1 (Cell Signaling Technology, Cat#47181, dilution 1:500). The tissues underwent a thorough washing process with PBS, repeated three times, before being treated with a secondary antibody solution for a duration of 2 h at room temperature. The secondary antibodies utilized in this research included goat anti‐rabbit 488 (Abcam, Cat#ab150077, diluted to 1:1000) and goat anti‐mouse 647 (Abcam, Cat#ab150115, also at 1:1000). Following the antibody incubation, the tissues were washed again with PBS, placed on glass slides, and then coverslipped using a mixture of glycerol and TBS in a 9:1 ratio, along with DAPI from Cell Signaling Technology. Imaging was carried out with a Nikon A1 confocal laser scanning microscope. The captured images were analyzed using ImageJ software (Fiji, NIH), with three sections examined per mouse to evaluate protein expression levels.

### 2.11 Enzyme‐Linked Immunosorbent Assay (ELISA)

The expression of IL‐1β, IL‐6, and TNF‐α was measured as we reported previously [32]. One day after the mice were exposed to sevoflurane, their amygdalae were meticulously extracted on ice. The collected tissue was subsequently homogenized in RIPA buffer (Sigma‐Aldrich), supplemented with a protease inhibitor cocktail (Sigma‐Aldrich). The levels of IL‐1β, IL‐6, and TNF‐α were measured using ELISA kits, following the manufacturer's guidelines closely (R&D Systems, MN).

### 2.12 TUNEL Staining

Following the manufacturer's guidelines (Promega, USA), we performed TUNEL staining to detect apoptotic cells in the amygdala 24 h post‐sevoflurane exposure. The brain slices underwent co‐staining with an anti‐NeuN monoclonal antibody (Abcam, Cat#ab104224, 1:500). Following this, the slices were treated with a biotinylated goat anti‐mouse antibody 647 for 1 h at room temperature. Imaging was performed utilizing a Zeiss 710 confocal microscope. The analysis of neurons exhibiting positive staining for NeuN and TUNEL in the mPFC and amygdala was conducted using Image J software (GE Healthcare, USA).

### 2.13 Statistical Analyses

All data analyses were conducted using GraphPad Prism 9.0 (USA). To examine the normality of the data distribution, the Shapiro–Wilk test was performed. The F‐test was utilized to check for homoscedasticity in datasets that followed a normal distribution. For data exhibiting non‐normal distributions, non‐parametric equivalents such as the Mann–Whitney *U* test or Kruskal‐Wallis test were applied. When comparing two independent groups with equal variances, an unpaired *t*‐test was employed. For scenarios involving multiple comparisons across more than two groups, a one‐way ANOVA followed by Tukey's post hoc test was implemented. In cases where two independent variables were analyzed, a two‐way repeated measures ANOVA with Bonferroni's post hoc test was employed. Data are presented as means ± SD, with a significance threshold set at *p* < 0.05.

## 3 Results

### 3.1 Sevoflurane Exposure Activated mPFC‐Amygdala Circuit and Induced Cognitive Deficits

**FIGURE 1:**
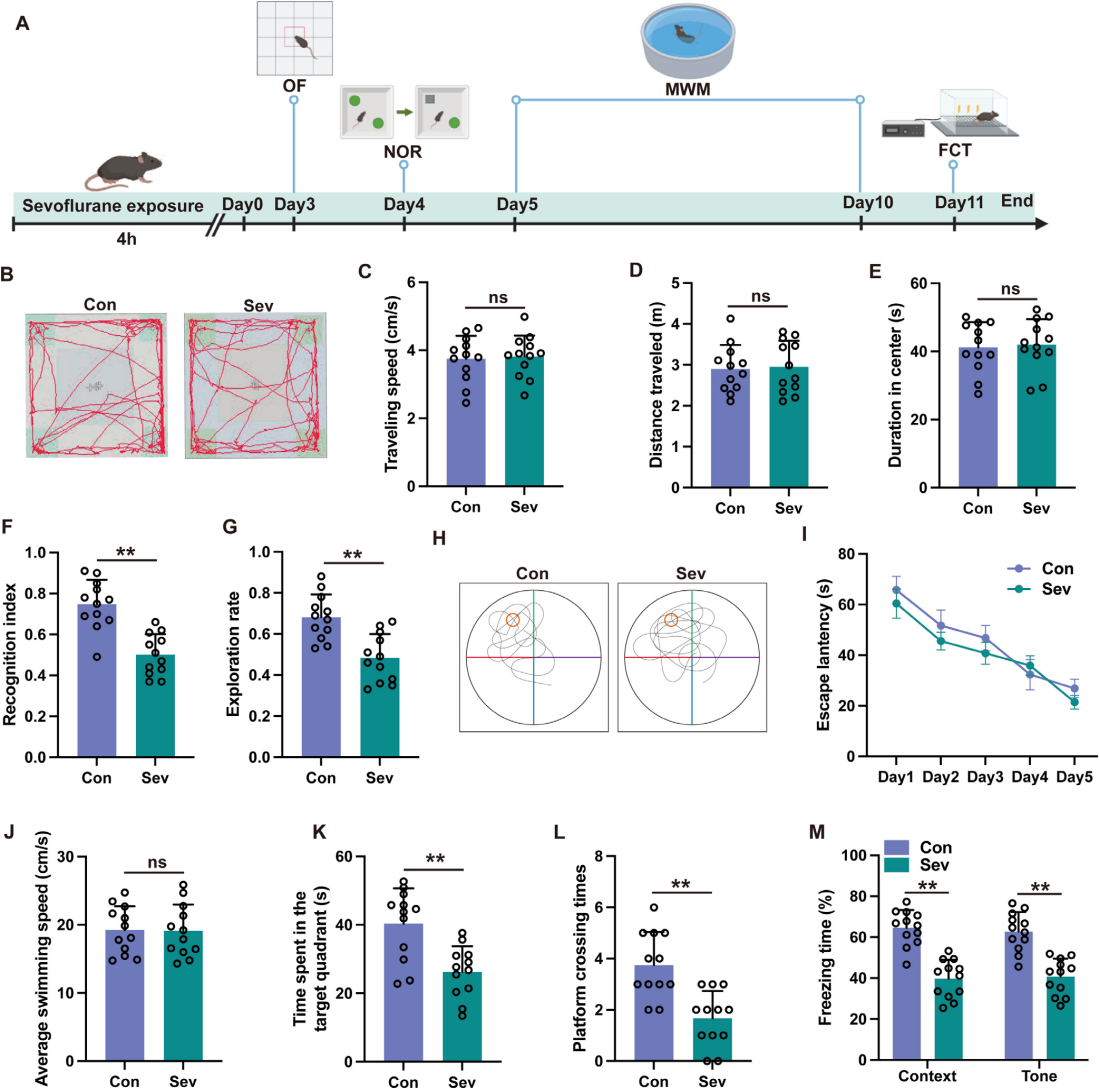
Sevoflurane exposure induced cognitive deficits. (A) Schematic diagram illustrating the timeline of experimental procedures in this study. (B) The representative trajectories of the aged mice in the open field test. (C–E) The traveling speed (C), distance traveled (D) and duration in the center (E) in the open field test. (F, G) The recognition index (F) and exploration rate (G) of the aged mice in the novel object recognition test. (H) The representative trajectories of the aged mice in the Morris water maze. (I–L) The escape latency (I), average swimming speed (J), time spent in the target quadrant (K) and platform crossing times (L) were recorded in the Morris water maze. (M) The contextual and cued freezing times were recorded in the fear conditioning test. Two‐way repeated measures ANOVA (panel I) and unpaired two‐tailed *t* tests (all other panels) were performed to evaluate the data. All data are presented as means ± SD. **p < 0.01, ns, not significant.

Initially, we investigated whether exposure to sevoflurane led to cognitive impairments in elderly mice. The schedule for the behavioral assessments is depicted in Figure 1A. In the open field test, there was no notable difference in the speed of movement, the total distance covered, or the time spent in the center zone between the Con group and Sev group (Figure 1B–E, *p* > 0.05), suggesting that the locomotor abilities of both groups were on par. Mice in the Sev group demonstrated a notably reduced recognition index (Figure 1F, *p* < 0.01) and exploration rate (Figure 1G, *p* < 0.01) when compared to the Con group during the novel object recognition test, suggesting that sevoflurane might lead to cognitive impairments. To evaluate spatial learning and memory capabilities of aged mice, the Morris water maze was employed. No significant disparities were detected in escape latency (Figure 1I, *p* > 0.05) or average swimming velocity (Figure 1J, *p* > 0.05) between the Con group and the Sev group during the spatial learning tests carried out 3–7 days following sevoflurane exposure. The findings reveal that spatial memory was effectively established following 5 days of training across all groups. Reference memory was evaluated through probe trials conducted 8 days after exposure to sevoflurane. Mice in the sevoflurane group spent notably less time in the target quadrant (Figure 1K, *p* < 0.01) and exhibited fewer crossings over the platform (Figure 1L, *p* < 0.01) when compared to the control group, indicating that sevoflurane caused a decline in reference memory. To assess the learning and memory abilities of the mice, a fear conditioning test was administered. The freezing response in the sevoflurane group was significantly reduced relative to the control group in both context and cued tests (Figure 1M, *p* < 0.01). Collectively, these outcomes highlight the extensive cognitive deficits associated with sevoflurane exposure.

**FIGURE 2:**
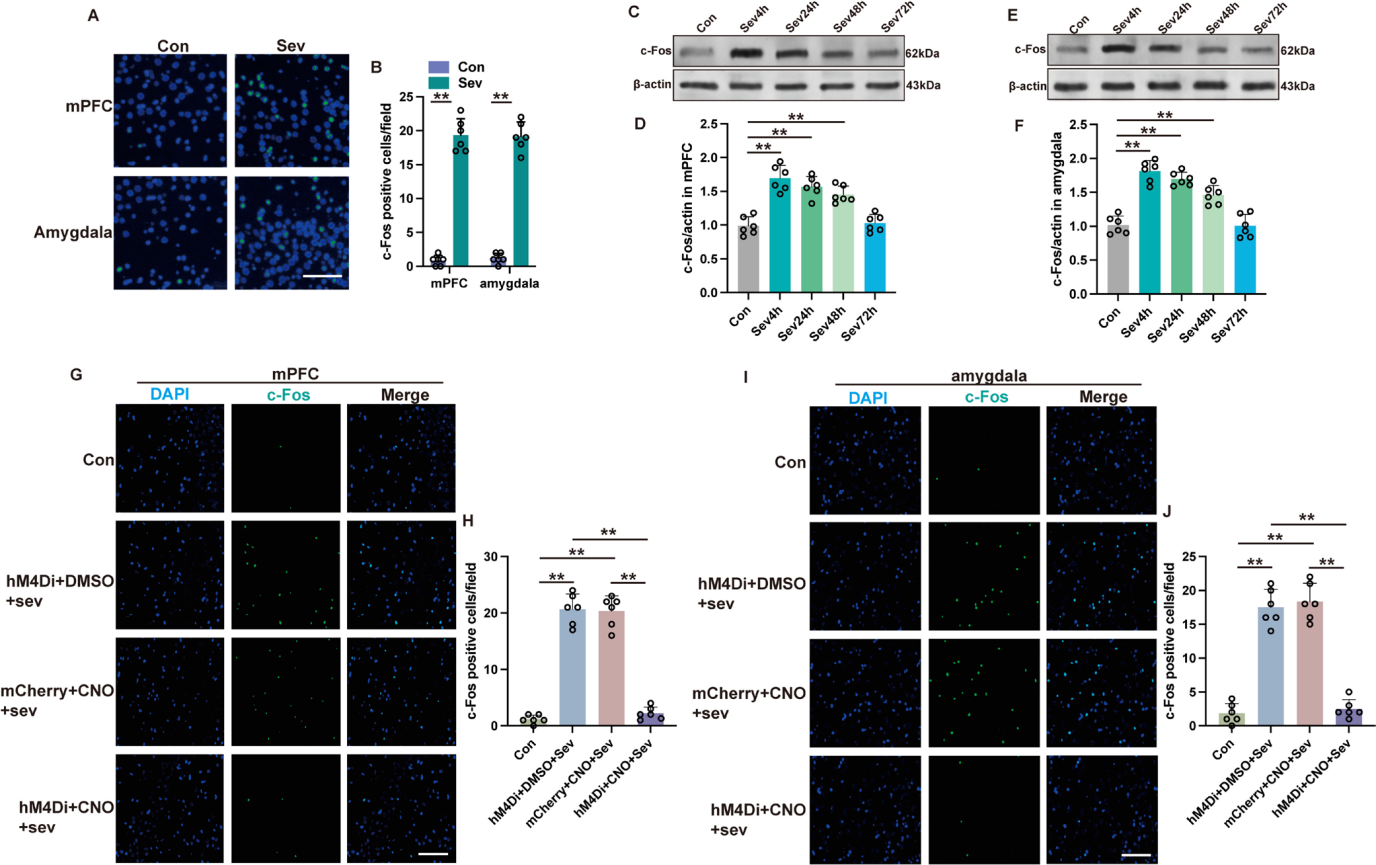
Sevoflurane exposure activated mPFC‐amygdala neuronal circuit. (A, B) The representative immunofluorescent images (A) and quantification (B) of c‐Fos positive cells at 4 h after sevoflurane exposure in mPFC and amygdala. Scale bar: 20 μm. (C, D) The representative western blot images (C) and quantification analysis (D) of c‐Fos expression in the mPFC at 4 h (Sev4h), 24 h (Sev24h), 48 h (Sev48h), or 72 h (Sev72h) after sevoflurane exposure. (E, F) The representative western blot images (E) and quantification analysis (F) of c‐Fos expression in the amygdala at 4 h (Sev4h), 24 h (Sev24h), 48 h (Sev48h), or 72 h (Sev72h) after sevoflurane exposure. (G, H) The representative immunofluorescent images (G) and quantification (H) of c‐Fos positive cells at 4 h after sevoflurane exposure in mPFC. Scale bar: 100 μm (I, J) The representative immunofluorescent images (I) and quantification (J) of c‐Fos positive cells at 4 h after sevoflurane exposure in amygdala. Scale bar: 100 μm. Results were analyzed by *t*‐test (panel B) and one‐way ANOVA followed by Tukey test (all other panels). All data are presented as means ± SD. **p < 0.01.

To elucidate the activation state of the mPFC‐amygdala circuit after sevoflurane exposure, immunofluorescence analysis for c‐Fos was conducted at 4 h after sevoflurane exposure. A pronounced elevation in c‐Fos‐positive cells was detected within the mPFC and amygdala regions (Figure 2A,B, *p* < 0.01), with sustained elevation in c‐Fos expression persisting at 24 h and 48 h after sevoflurane exposure (Figure 2C–F, *p* < 0.01) and returning to the baseline at 72 h after sevoflurane exposure (Figure 2C–F, *p* > 0.05). Projection verification from the mPFC to the amygdala was achieved through injection of AAV2‐hSyn‐mCherry into the mPFC. Three weeks after the injection, follow‐up confocal microscopy showed the connections extending from the mPFC to the amygdala. The projection fibers originating from the mPFC were found to co‐localize with vesicular glutamate transporter 1 (VGLUT1), while showing no overlap with the vesicular GABA transporter (VGAT) (refer to Figure S1). These data suggest that sevoflurane activated the mPFC‐amygdala circuit and that the projections from the mPFC to the amygdala are predominantly glutamatergic.

### 3.2 Suppression of mPFC‐Amygdala Circuit Mitigates Sevoflurane‐Induced Cognitive Deficits

**FIGURE 3:**
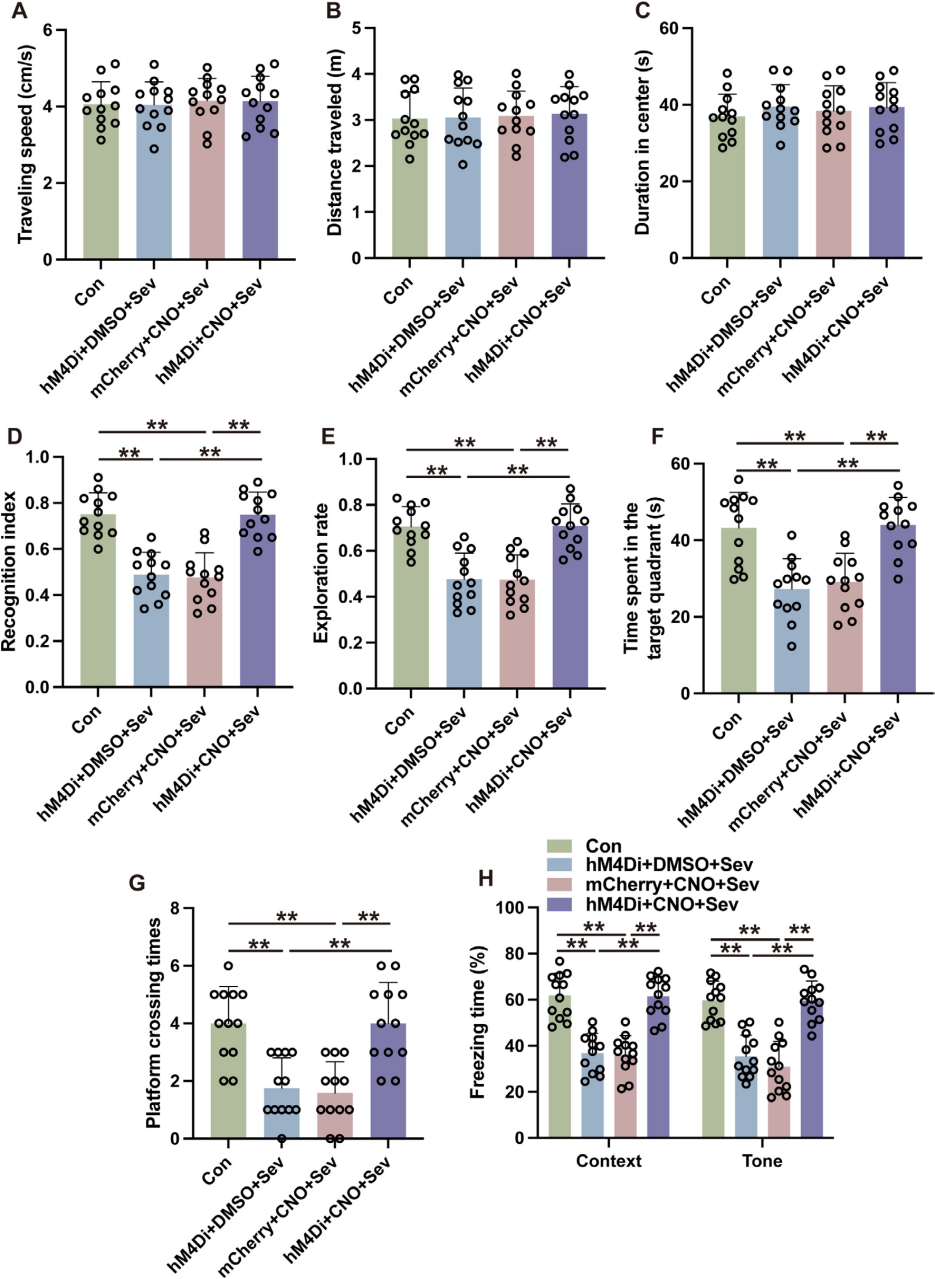
Suppression of mPFC‐amygdala circuit mitigates sevoflurane‐induced cognitive deficits. (A–C) The traveling speed (A), distance traveled (B) and duration in the center (C) in the open field test. (D, E) The recognition index (D) and exploration rate (E) of the aged mice in the novel object recognition test. (F, G) The time spent in the target quadrant (F) and platform crossing times (G) were recorded in the Morris water maze. (H) The contextual and cued freezing times were recorded in the fear conditioning test. Results were analyzed by one‐way ANOVA followed by Tukey test. All data are presented as means ± SD. **p < 0.01.

The role of the mPFC‐amygdala circuit in the development of cognitive impairments caused by sevoflurane was investigated by employing a chemogenetic approach to suppress neuronal activity in the mPFC. The mPFC received viral vectors carrying either hM4Di‐mCherry conjugated to a control vector (AAV2‐hSyn‐mCherry) or the human synapsin promoter (AAV2‐hSyn‐hM4Di‐mCherry). Successful transduction was achieved in over 80% of mPFC neurons (refer to Figure S2). In mice exposed to sevoflurane, the introduction of CNO alongside AAV2‐hSyn‐hM4Di‐mCherry led to a notable decrease in c‐Fos expression within the mPFC (Figure 2G,H, *p* < 0.01) and the amygdala (Figure 2I,J, *p* < 0.01). This reduction suggests a drop in neural activity compared to mice that were also exposed to sevoflurane but received either AAV2‐hsyn‐hM4Di‐mCherry combined with DMSO or AAV2‐hsyn‐mCherry with CNO. The density of c‐Fos immunoreactive neurons in mice exposed to sevoflurane and subsequently treated with AAV2‐hSyn‐hM4Di‐mCherry in combination with DMSO was comparable to the baseline levels observed in the control group, which underwent neither sevoflurane exposure nor received any injections (Figure 2G–J, *p* > 0.05). These findings indicate that the utilization of chemogenetic constructs successfully mitigates the activity within the mPFC‐amygdala circuit following sevoflurane exposure. Inhibition of mPFC activity did not translate to alterations in locomotor activity in mice exposed to sevoflurane, as evidenced by unchanged results in the open field test (Figure 3A–C, *p* > 0.05). However, the identical inhibitory treatment significantly enhanced the recognition index (Figure 3D, *p* < 0.01) and exploration rate (Figure 3E, *p* < 0.01) in mice subjected to sevoflurane. The findings from the MWM suggest that inhibiting the mPFC significantly prolonged the duration that mice spent in the target quadrant (Figure 3F, *p* < 0.01) and increased the frequency of platform crossings (Figure 3G, *p* < 0.01) in those subjected to sevoflurane. Furthermore, this suppressive effect was linked to a heightened freezing response during both contextually and tonally cued fear conditioning tests (Figure 3H, *p* < 0.01). Taken together, these results point to mPFC activation as a key factor underlying the cognitive and memory impairments that arise after exposure to sevoflurane.

**FIGURE 4:**
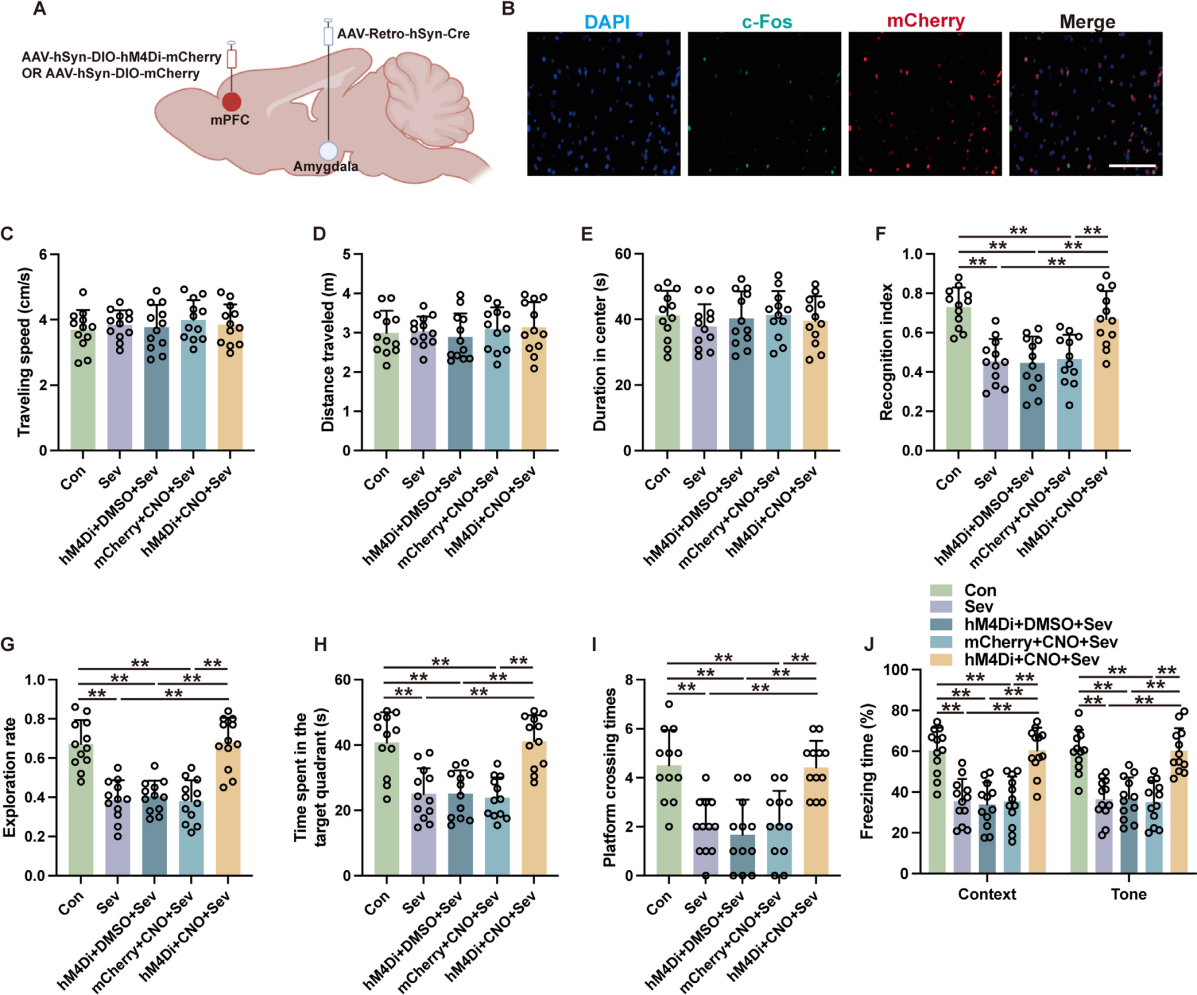
Retrograde inhibition of mPFC neurons via the projections from mPFC to amygdala ameliorated sevoflurane‐induced cognitive deficits. (A) Schematic of the retrograde tracing virus injections. (B) Representative immunofluorescent images of hM4Di‐transduced neurons and c‐Fos expression following sevoflurane exposure and intraperitoneal DMSO injection. Scale bar: 20 μm. (C–E) The traveling speed (C), distance traveled (D), and duration in the center (E) in open field test. (F, G) The recognition index (F) and exploration rate (G) of the aged mice in the novel object recognition test. (H, I) The time spent in the target quadrant (H) and platform crossing times (I) were recorded in the Morris water maze. (J) The contextual and cued freezing times were recorded in the fear conditioning test. Results were analyzed by one‐way ANOVA followed by Tukey test. All data are presented as means ± SD. **p < 0.01.

In the pursuit of elucidating the involvement of the mPFC‐amygdala circuit in sevoflurane‐induced cognitive deficits, mice received an AAV‐Retro‐hSyn‐Cre injection into the amygdala, paired with either AAV‐hSyn‐DIO‐hM4Di‐mCherry or AAV‐hSyn‐DIO‐mCherry in the mPFC (Figure 4A,B). The results of the open field test revealed no significant differences in locomotor and exploratory behavior among the five distinct experimental groups subjected to varied treatments (Figure 4C–E, *p* > 0.05). Nonetheless, mice that underwent sevoflurane exposure coupled with retrograde mPFC inhibition exhibited a significantly higher recognition index (Figure 4F, *p* < 0.01) and exploration rate (Figure 4G, *p* < 0.01) in contrast to the mice that underwent sevoflurane without experiencing neuronal inhibition. Moreover, the MWM performance metrics indicated that retrograde inhibition of mPFC neurons notably elevated the duration spent in the target quadrant (Figure 4H, *p* < 0.01) as well as the frequency of platform crossings (Figure 4I, *p* < 0.01) in mice subjected to sevoflurane. Furthermore, such inhibition elicited an augmentation in the freezing response during both contextually and tonally cued fear conditioning tests in mice exposed to sevoflurane (Figure 4J, *p* < 0.01). Collectively, these findings underscore the critical role of the mPFC‐amygdala circuit in the etiology of learning and memory impairments triggered by sevoflurane.

### 3.3 Provocation of mPFC Neuronal Activity Induced Cognitive Impairments

**FIGURE 5:**
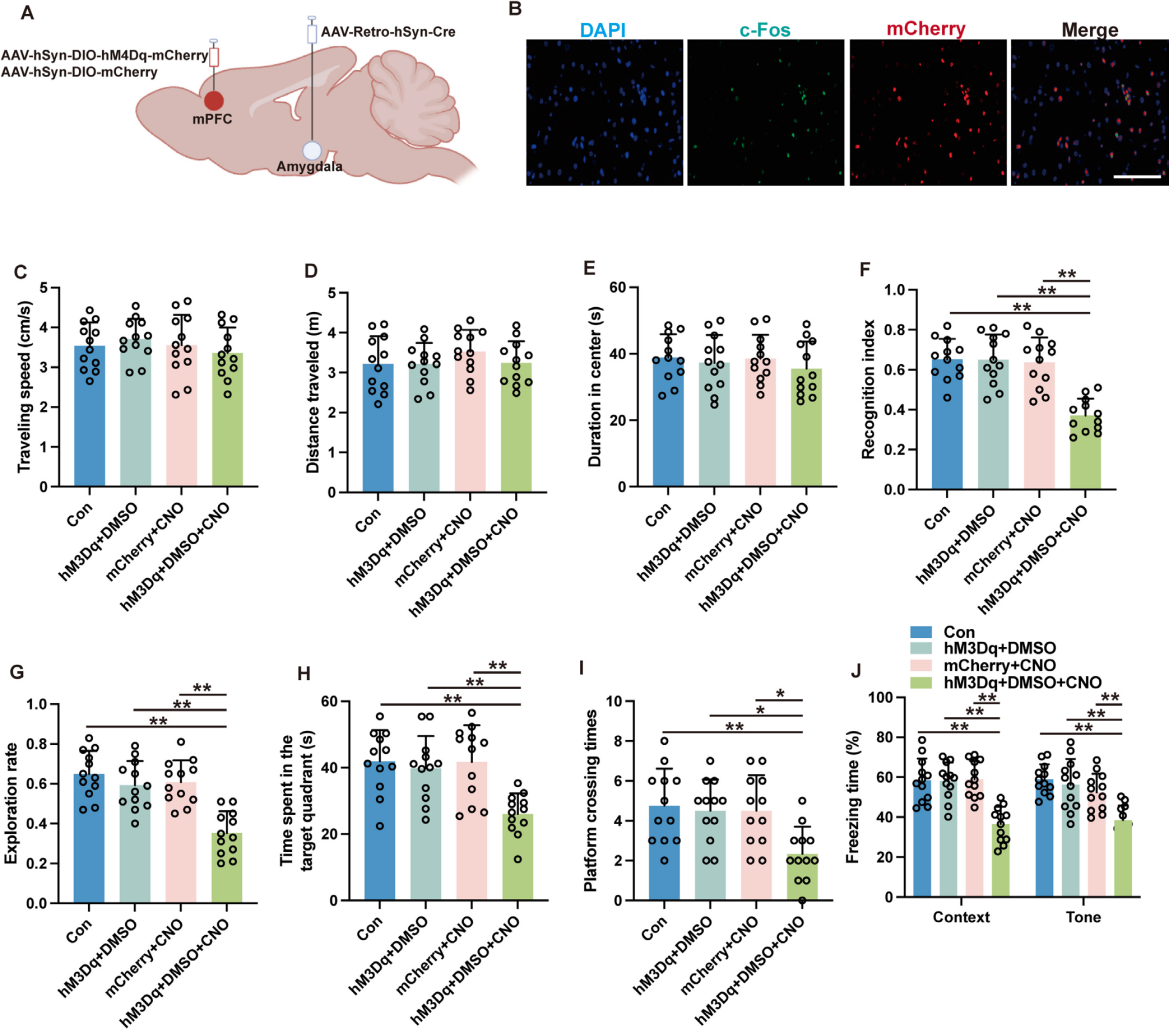
Chemogenetic activation of the mPFC‐amygdala neuronal circuit induced cognitive deficits. (A) Schematic of the retrograde tracing virus injections. (B) Representative immunofluorescent images of hM4D q‐transduced neurons and c‐Fos expression following sevoflurane exposure and intraperitoneal DMSO injection. Scale bar: 20 μm. (C–E) The traveling speed (C), distance traveled (D), and duration in the center (E) in the open field test. (F, G) The recognition index (F) and exploration rate (G) of the aged mice in the novel object recognition test. (H, I) The time spent in the target quadrant (H) and platform crossing times (I) were recorded in the Morris water maze. (J) The contextual and cued freezing times were recorded in the fear conditioning test. Results were analyzed by one‐way ANOVA followed by Tukey test. All data are presented as means ± SD. *p < 0.05, **p < 0.01.

**FIGURE 6:**
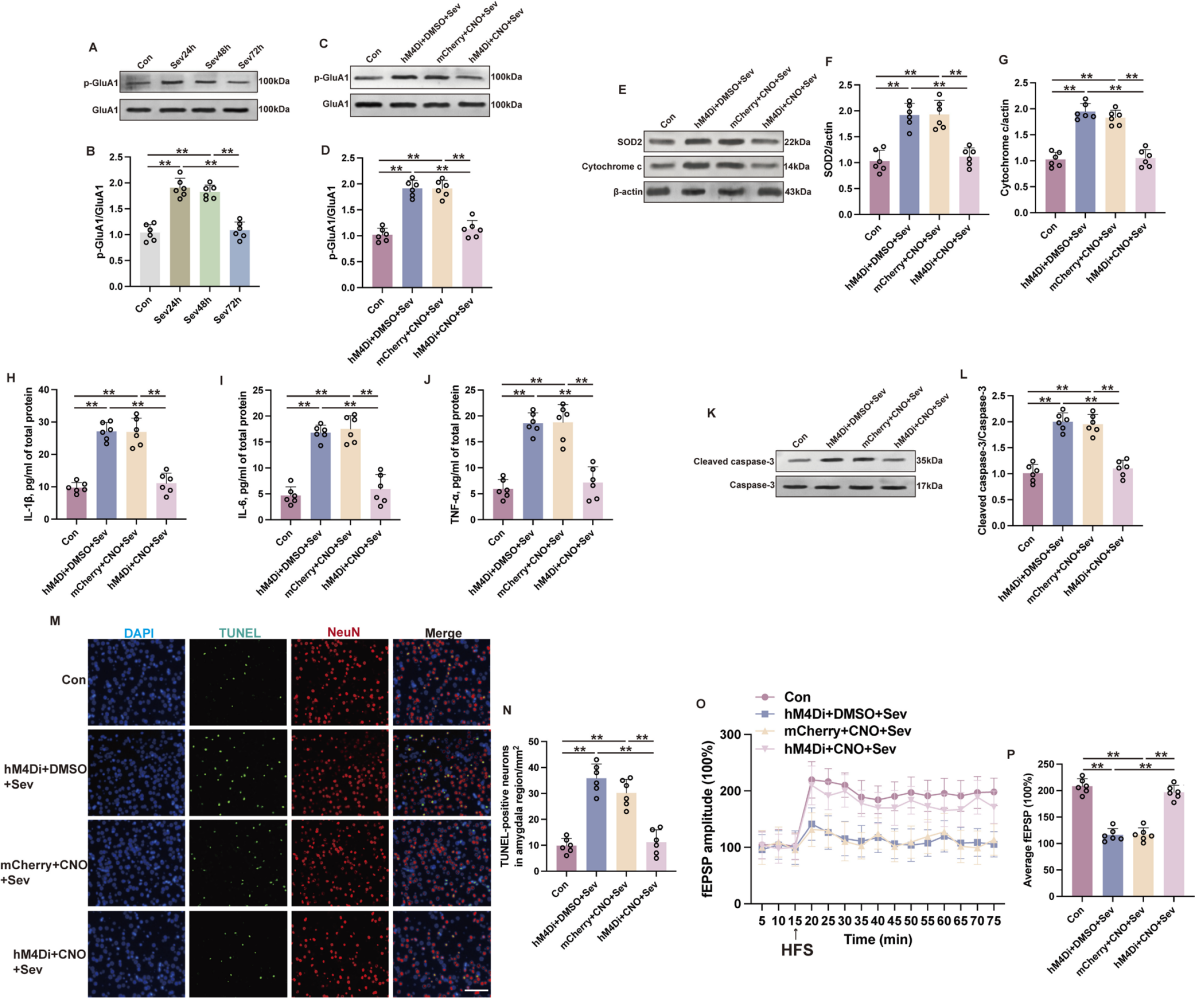
Inhibition of the mPFC‐amygdala neuronal circuit alleviated sevoflurane‐induced AMPA receptor activation and mitochondrial stress in aged mice. (A, B) The representative western blot images (A) and quantification analysis (B) of p‐GluA1/GluA1 in the amygdala at 24 h (Sev24h), 48 h (Sev48h), or 72 h (Sev72h) after sevoflurane exposure. (C, D) The representative western blot images (C) and quantification analysis (D) of p‐GluA1/GluA1 in the amygdala at 24 h after sevoflurane exposure. (E–G) The representative western blot images (E) and quantification analysis of SOD2 (F) and cytochrome c (G) in the amygdala at 24 h after sevoflurane exposure. (H–J) Expressions of IL‐1β (H), IL‐6 (I), and TNF‐α (J) in the amygdala at 24 h after sevoflurane exposure were detected by ELISA. (K, L) The representative western blot images (K) and quantification analysis of cleaved caspase‐3/caspase‐3 (L) in the amygdala at 24 h after sevoflurane exposure. (M, N) The representative immunofluorescent images (M) and quantification (N) of TUNEL‐positive neurons in the amygdala at 24 h after sevoflurane exposure. Scale bar: 100 μm. (O) The change of fEPSP slope over time in the amygdala at 24 h after sevoflurane exposure. (P) The quantitative analysis of the mean fEPSP slope at the 70th min. Results were analyzed by one‐way ANOVA followed by Tukey test. All data are presented as means ± SD. **p < 0.01.

To trigger retrograde activation of mPFC neurons, mice received an injection of AAV‐Retro‐hSyn‐Cre into the amygdala, along with either AAV‐hSyn‐DIO‐hM3Dq‐mCherry or AAV‐hSyn‐DIO‐mCherry into the mPFC (Figures 5A and 6B). No detectable changes in locomotion were observed in the open field test despite the retrograde activation of mPFC neurons (Figure 5C–E, *p* > 0.05). In contrast, the recognition index (Figure 5F, *p* < 0.01) and exploration rate (Figure 5G, *p* < 0.01) were reduced in mice subjected to retrograde neuronal activation within the mPFC when compared to control mice or those receiving viral vectors without subsequent activation of mPFC neurons by CNO. During MWM, the induction of retrograde neuronal activation within mPFC was found to reduce the time spent in the target quadrant (Figure 5H, *p* < 0.01) and platform crossing times (Figure 5I, *p* < 0.05) when compared with the mCherry + CNO group and the hM3Dq + CNO group. Moreover, this same activation resulted in a diminution of freezing behavior in mice exposed to sevoflurane during contextually and tonally cued fear conditioning tests (Figure 5J, *p* < 0.01). Collectively, these observations endorse the hypothesis that activation of mPFC neurons, via the mPFC‐amygdala circuit, is capable of inducing deficits in learning and memory ability.

### 3.4 Sevoflurane Prompts Mitochondrial Stress in the Amygdala by Activating the AMPA Receptor

An investigation into the molecular changes within the amygdala was conducted to explore the underlying mechanism after the activation of the mPFC‐amygdala circuit for PND. The phosphorylation state of AMPA receptor 1 (p‐GluA1‐Ser831) within the amygdala exhibited an elevation at 24 h and 48 h following sevoflurane exposure, normalizing to baseline levels at 72 h after sevoflurane exposure (Figure 6A,B). This pattern corresponds with the activation timeline of the mPFC‐amygdala circuit. Application of DREADDs to inhibit mPFC activity resulted in a reduction of p‐GluA1 within the mPFC (Figure 6C,D, *p* < 0.01). Given the predominantly glutamatergic nature of mPFC projections to the amygdala and the established correlation between p‐GluA1 levels and AMPA receptor activity, alongside the established link between sustained AMPA receptor activation and excitotoxicity within the amygdala, an assessment of mitochondrial stress was thus conducted. Mitochondrial stress‐associated protein expression, specifically superoxide dismutase 2 (SOD2) and cytochrome c, was observed to be mitigated within the amygdala in the Sev group that received mPFC inhibition, compared with mice in the Sev group without such mPFC inhibition (Figure 6E–G, *p* < 0.01). Furthermore, this inhibition of mPFC neural activity coincided with a reduction in levels of inflammatory cytokines, including IL‐6, IL‐1β, and TNF‐α (Figure 6H–J, *p* < 0.01) as well as an attenuated presence of cleaved caspase‐3 (Figure 6K,L, *p* < 0.01), a pivotal effector of apoptosis, within the amygdala after sevoflurane exposure. Additionally, a commensurate decline in neuronal apoptosis within the amygdala was observed (Figure 6M,N, *p* < 0.01), as indicated by reduced terminal deoxynucleotidyl transferase dUTP nick end labeling (TUNEL) positive cells in the Sev group with mPFC inhibition. It is pertinent to note that the amygdala is a neural region densely populated by glutamatergic neurons [33] and the amygdala sends glutamatergic projections to the mPFC, with glutamate playing a critical role in modulating LTP—the main mechanisms underlying learning and memory formation [34, 35]. As indicated by our previous study, sevoflurane exposure may induce LTP suppression [29, 36]. Aligning with our previous conclusions, the attenuation of mPFC‐amygdala circuitry activity was found to ameliorate LTP suppression within the amygdala in mice exposed to sevoflurane (Figure 6O,P, *p* < 0.01). Collectively, these findings intimate that the mPFC‐amygdala circuit activation may aggravate sevoflurane‐induced mitochondrial stress, neuroinflammatory cascades, cellular damage, and LTP suppression.

### 3.5 Inhibiting Amygdala AMPA Receptor Activity Mitigates Sevoflurane‐Induced Cognitive Dysfunction via Mitochondrial Stress Attenuation

**FIGURE 7:**
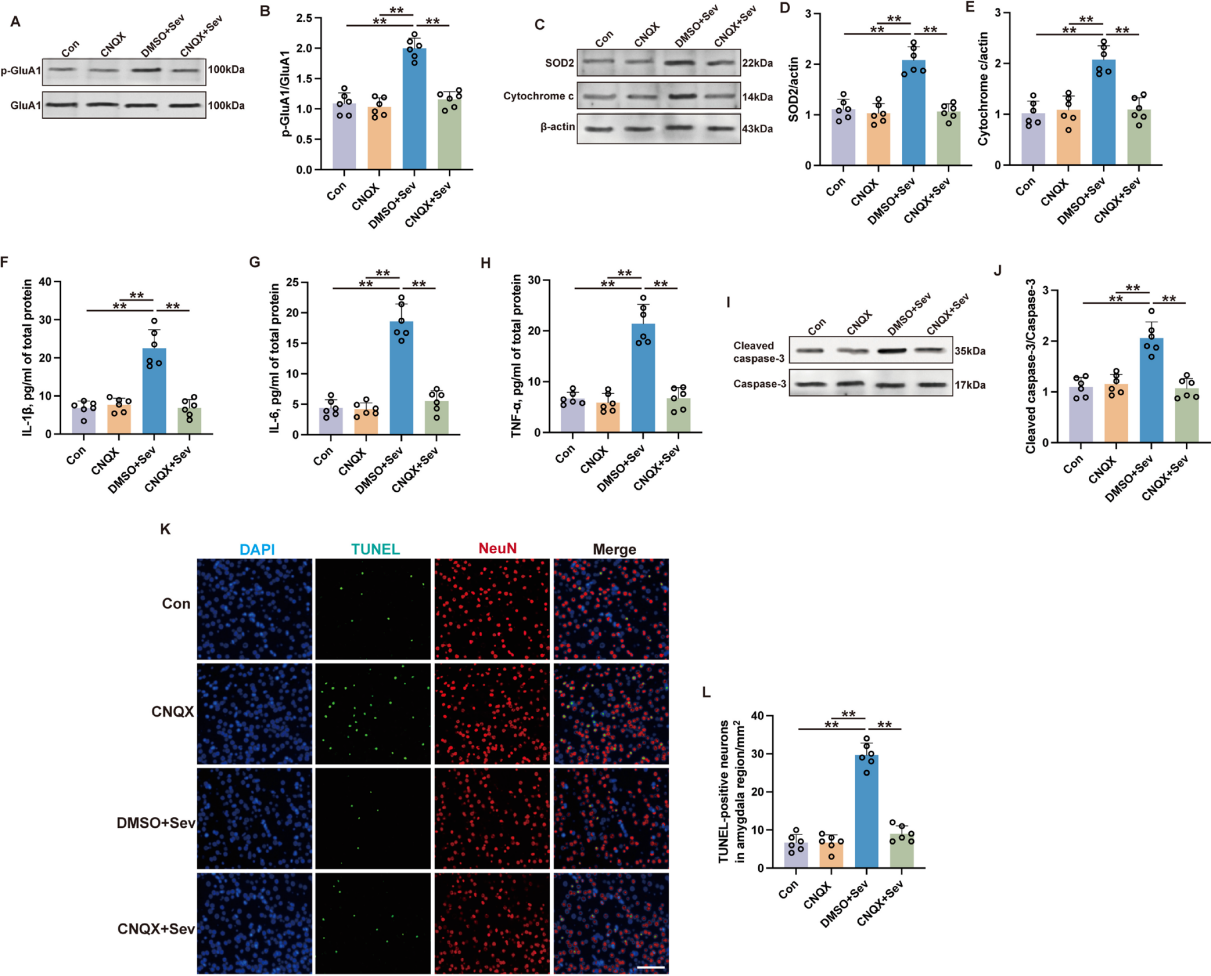
Inhibition of AMPA receptors by CNQX mitigated sevoflurane‐induced mitochondrial stress and neuropathological alterations in aged mice. (A, B) The representative western blot images (A) and quantification analysis (B) of p‐GluA1/GluA1 in the amygdala at 24 h after sevoflurane exposure. (C–E) The representative western blot images (C) and quantification analysis of SOD2 (D) and cytochrome c (E) in the amygdala at 24 h after sevoflurane exposure. (F–H) Expressions of IL‐1β (F), IL‐6 (G), and TNF‐α (H) in the amygdala at 24 h after sevoflurane exposure were detected by ELISA. (I, J) The representative western blot images (I) and quantification analysis of cleaved caspase‐3/caspase‐3 (J) in the amygdala at 24 h after sevoflurane exposure. (K, L) The representative immunofluorescent images (K) and quantification (L) of TUNEL‐positive neurons in the amygdala at 24 h after sevoflurane exposure. Scale bar: 100 μm. Results were analyzed by one‐way ANOVA followed by Tukey test. All data are presented as means ± SD. **p < 0.01.

As demonstrated above, sevoflurane was observed to upregulate the expression of p‐GluA1, indicative of enhanced AMPA receptor activation. The use of 6‐Cyano‐7‐nitroquinoxaline‐2,3‐dione (CNQX), an AMPA receptor antagonist, within the amygdala was associated with a reduction in the expression levels of p‐GluA1. This intervention also led to a reduction in SOD2, cytochrome c, IL‐6, TNF‐α, IL‐1β, and cleaved caspase‐3 following sevoflurane exposure (Figure 7A–J). Moreover, CNQX application was correlated with a decrease in neuronal apoptosis (Figure 7K,L). This evidence collectively indicates that antagonism of NMDA receptor signaling within the amygdala may serve as a neuroprotective strategy against cognitive impairments following sevoflurane exposure, potentially by mitigating the deleterious effects of mitochondrial stress and subsequent neuronal apoptosis.

**FIGURE 8:**
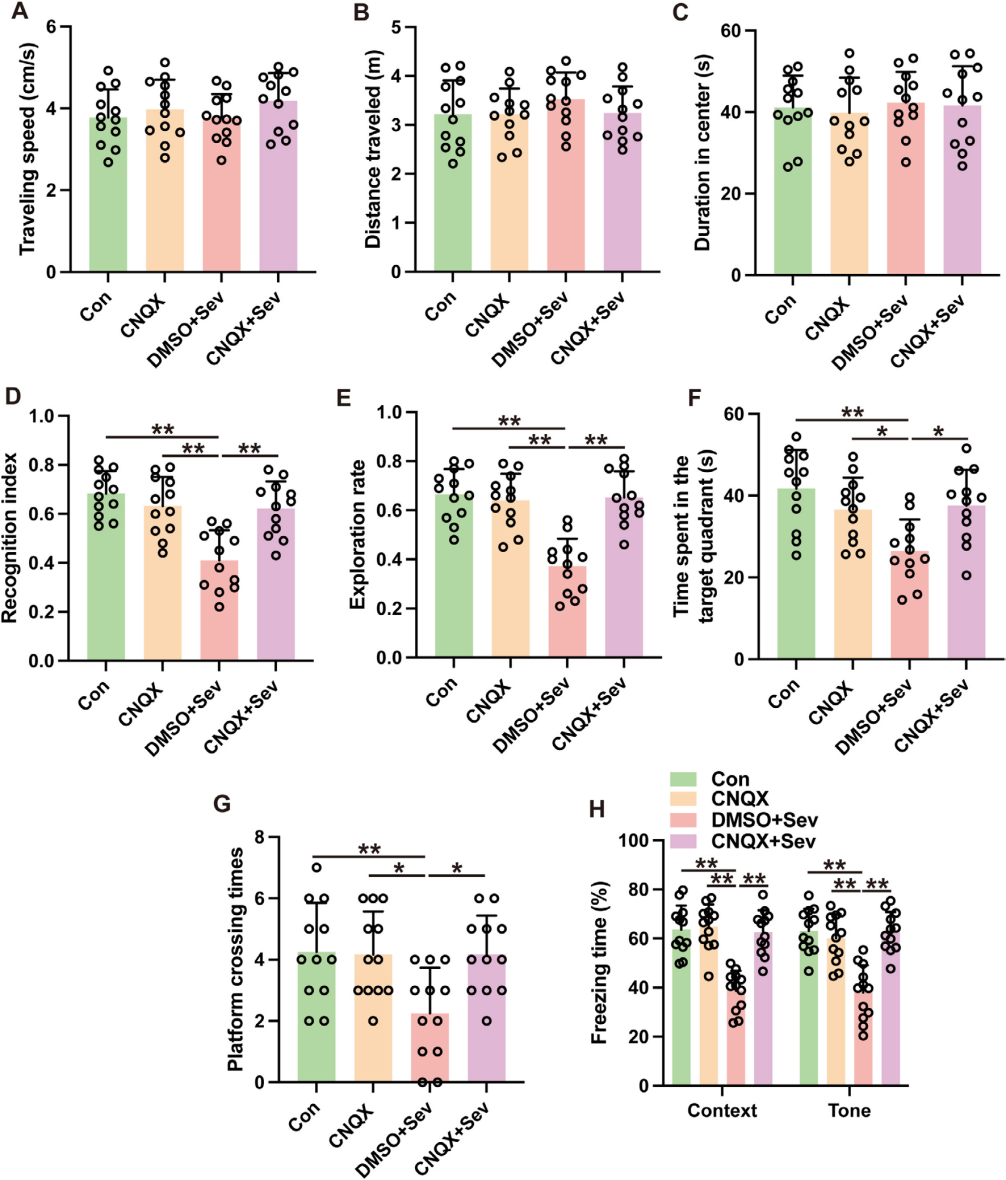
Inhibition of AMPA receptors by CNQX mitigated sevoflurane‐induced cognitive deficits in aged mice. (A–C) The traveling speed (A), distance traveled, (B) and duration in the center (C) in open field test. (D, E) The recognition index (D) and exploration rate (E) of the aged mice in the novel object recognition test. (F, G) The time spent in the target quadrant (F) and platform crossing times (G) were recorded in the Morris water maze. (H) The contextual and cued freezing times were recorded in the fear conditioning test. Results were analyzed by one‐way ANOVA followed by Tukey test. All data are presented as means ± SD. *p < 0.05, **p < 0.01.

Intriguingly, the administration of CNQX to the amygdala kept the mice's baseline activity steady during the open field test (Figure 8A–C, *p* > 0.05), yet it seemed to boost their cognitive abilities, particularly in those mice subjected to sevoflurane, as indicated by their impressive performance in the novel object recognition task (Figure 8D,E, *p* < 0.01). Furthermore, CNQX administration improved the cognitive performance of mice in the Sev group during MWM. In comparison to the DMSO+Sev group, the CNQX + Sev group showed a significant increase in time spent in the target quadrant (Figure 8F, *p* < 0.05) and in platform crossing times (Figure 8G, *p* < 0.01). CNQX administration led to a heightened freezing response in rodents subjected to sevoflurane during both contextually and tonally prompted fear conditioning assessments (see Figure 8H, *p* < 0.01). This suggests that the activation of AMPA receptors within the amygdala plays a pivotal role in the neuropathological changes and resulting cognitive impairments in learning and memory observed in mice post‐sevoflurane exposure.

### 3.6 Role of Amygdala Glutamatergic Neuronal Activity in Sevoflurane‐Induced Cognitive Impairments

**FIGURE 9:**
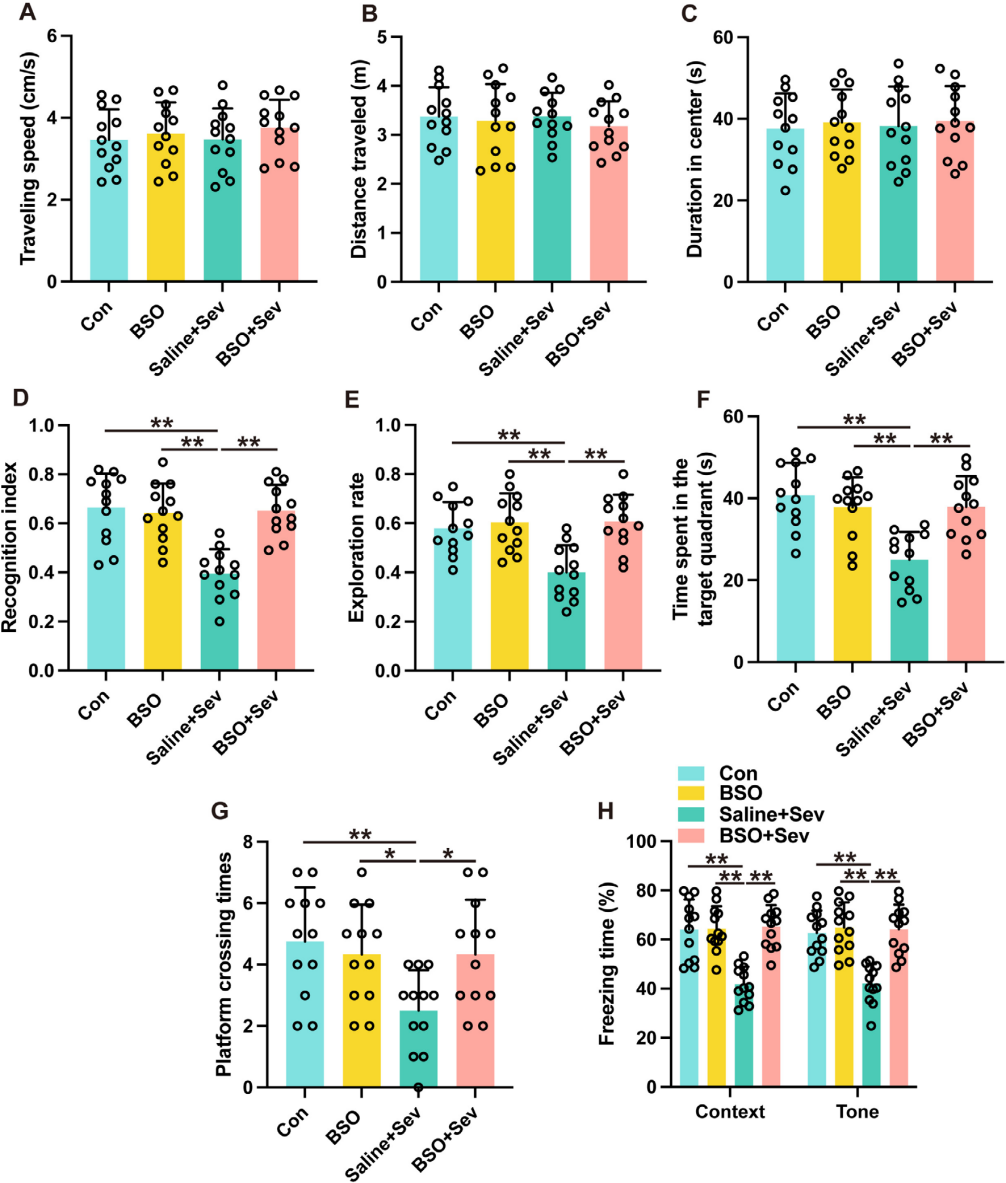
Inhibition of glutaminase by BSO ameliorated sevoflurane‐induced cognitive deficits in aged mice. (A–C) The traveling speed (A), distance traveled, (B) and duration in the center (C) in open field test. (D, E) The recognition index (D) and exploration rate (E) of the aged mice in the novel object recognition test. (F, G) The time spent in the target quadrant (F) and platform crossing times (G) were recorded in the Morris water maze. (H) The contextual and cued freezing times were recorded in the fear conditioning test. Results were analyzed by one‐way ANOVA followed by Tukey test. All data are presented as means ± SD. *p < 0.05, **p < 0.01.

An examination into the role of amygdala glutamatergic neurons in sevoflurane's impact on cognitive impairments was conducted by administering buthionine sulfoximine (BSO), a substance that hampers the activity of glutaminase—an enzyme essential for the production of glutamate in neurons. The administration of BSO into the amygdala did not alter locomotive behavior in the open field test, indicating no effect on general activity (Figure 9A–C, *p* > 0.05). Notwithstanding, BSO administration ameliorated sevoflurane‐induced cognitive deficits in the novel object recognition test (Figure 9D,E, *p* < 0.01). Additionally, BSO treatment ameliorated the performance of mice exposed to sevoflurane in MWM. Mice that were subjected to sevoflurane and subsequently treated with BSO showed a marked increase in the duration spent in the target quadrant (Figure 9F, *p* < 0.01) as well as a higher number of platform crossings (Figure 9G, *p* < 0.05) compared to those exposed to sevoflurane alone without BSO treatment. Furthermore, the BSO treatment resulted in a heightened freezing reaction among mice subjected to sevoflurane during both contextually and tonally cued fear conditioning trials (Figure 9H, *p* < 0.01). These findings suggest that glutamatergic neurons in the amygdala significantly contribute to the development of learning and memory impairments caused by sevoflurane.

### 3.7 Suppression of Mitochondrial Stress With Amygdala Mitigated Sevoflurane‐Induced Cognitive Deficits

**FIGURE 10:**
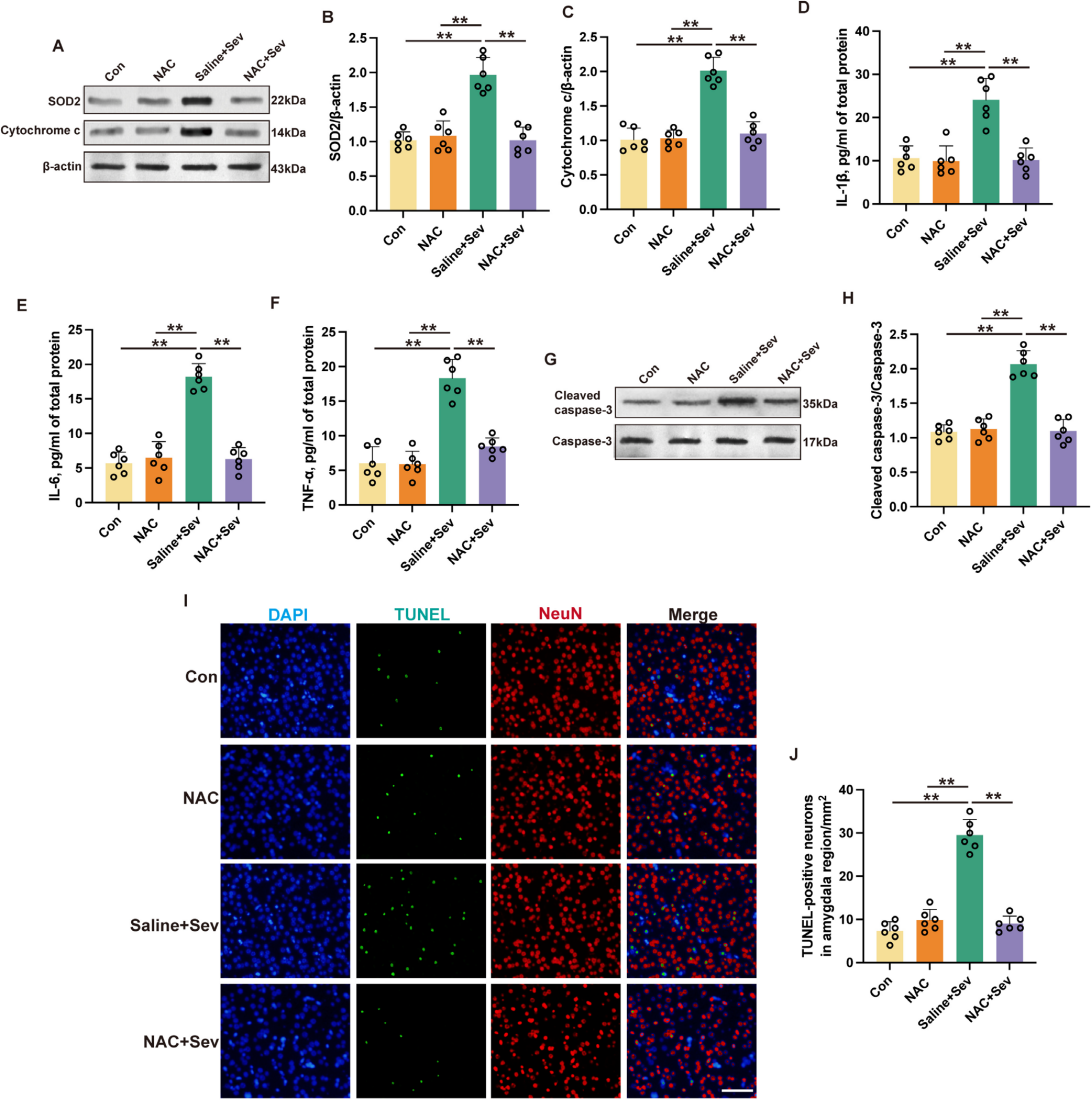
Inhibition of mitochondrial stress by NAC attenuated sevoflurane‐induced neuronal apoptosis and inflammatory responses in aged mice. (A–C) The representative western blot images (A) and quantification analysis of SOD2 (B) and cytochrome c (C) in the amygdala at 24 h after sevoflurane exposure. (D–F) Expressions of IL‐1β (D), IL‐6 (E), and TNF‐α (F) in the amygdala at 24 h after sevoflurane exposure were detected by ELISA. (G, H) The representative western blot images (G) and quantification analysis of cleaved caspase‐3/caspase‐3 (H) in the amygdala at 24 h after sevoflurane exposure. (I, J) The representative immunofluorescent images (I) and quantification (J) of TUNEL‐positive neurons in the amygdala at 24 h after sevoflurane exposure. Scale bar: 100 μm. Results were analyzed by one‐way ANOVA followed with Tukey test. All data are presented as means ± SD. **p < 0.01.

**FIGURE 11:**
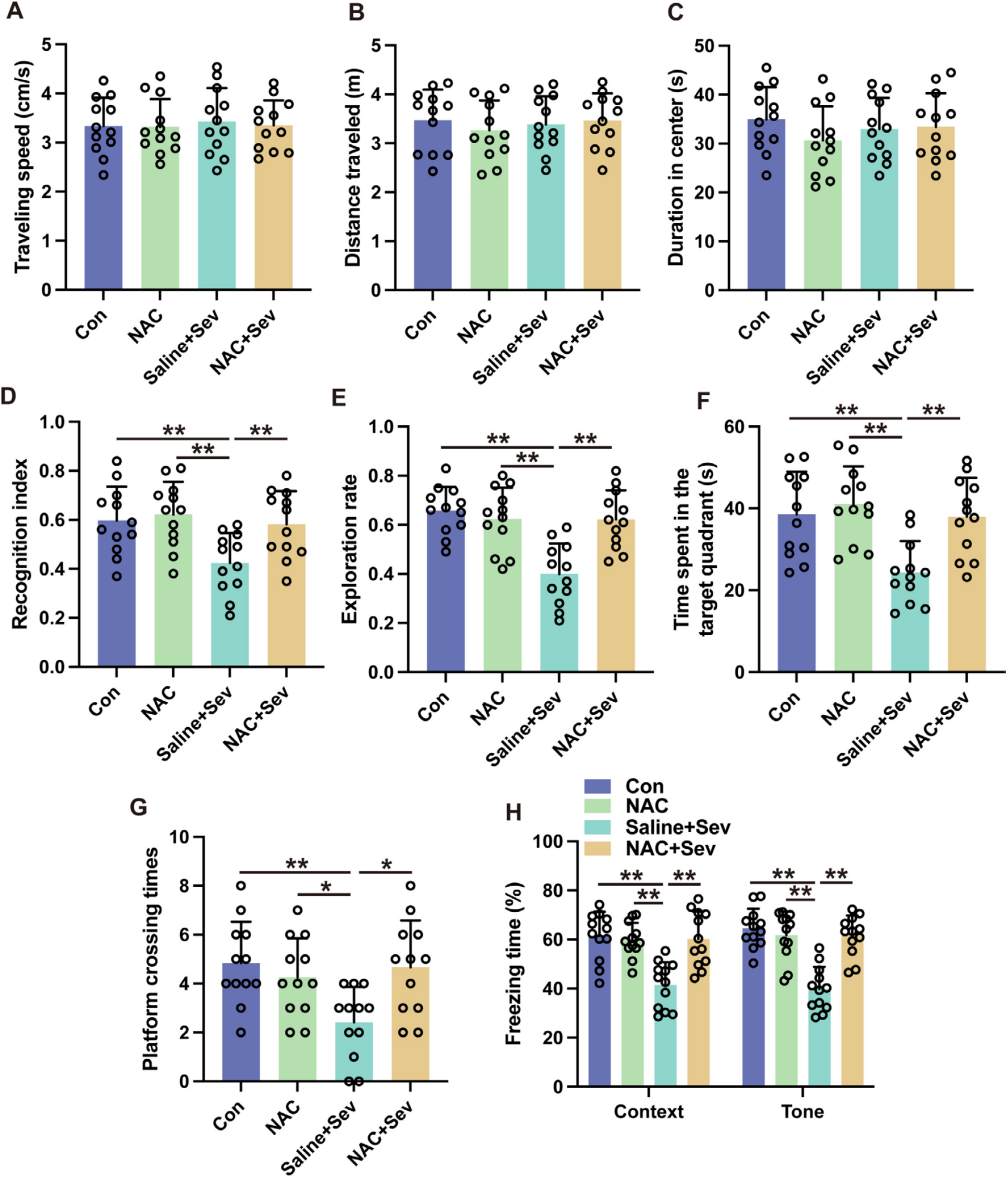
Inhibition of mitochondrial stress by NAC attenuated sevoflurane‐induced cognitive deficits in aged mice. (A–C) The traveling speed (A), distance traveled (B), and duration in the center (C) in open field test. (D, E) The recognition index (D) and exploration rate (E) of the aged mice in novel object recognition test. (F, G) The time spent in the target quadrant; (F) platform crossing times (G) were recorded in Morris water maze. (H) The contextual and cued freezing times were recorded in fear conditioning test. Results were analyzed by one‐way ANOVA followed by Tukey test. All data are presented as means ± SD. *p < 0.05, **p < 0.01.

To clarify how mitochondrial stress contributes to the development of cognitive impairments associated with sevoflurane, N‐acetylcysteine (NAC), which acts as a mitochondrial stress inhibitor, was administered directly into the amygdala. Subsequent to treatment, a downregulation in the expression levels of SOD2 and cytochrome c, biomarkers of mitochondrial stress, was observed after sevoflurane exposure (Figure 10A–C, *p* < 0.01). Furthermore, NAC administration decreased the level of TNF‐α, IL‐6, IL‐1β, and cleaved caspase‐3 within the amygdala of sevoflurane‐exposed mice (Figure 10D–H, *p* < 0.01). NAC administration resulted in a diminution of apoptotic cellular profiles as assessed by TUNEL staining (Figure 10I,J, *p* < 0.01). While NAC's influence on locomotor activity of mice exposed to sevoflurane was negligible (Figure 11A–C, *p* > 0.05), it notably enhanced the duration of novel object exploration in mice exposed to sevoflurane (Figure 11D,E, *p* < 0.01). Additionally, NAC conferred improvements in cognitive performance as evidenced by MWM (Figure 11F,G, *p* < 0.05). An elevation in conditioned freezing responses was also observed in both contextually and tonally cued fear conditioning paradigms following NAC treatment in mice exposed to sevoflurane (Figure 11H, *p* < 0.01). Collectively, these findings implicate mitochondrial stress within the amygdala as a modulator of the inflammatory milieu, cellular demise, and the disruption of cognitive function ensuing sevoflurane exposure.

## 4 Discussion

The precise neurocircuitry underlying PND, a prominent clinical manifestation during the perioperative period, remains elusive. The findings from our investigation indicate that sevoflurane induces activation of the mPFC‐amygdala neural circuit. Moreover, interventions that disrupted this circuit mitigated the cognitive deficits induced by sevoflurane. This study serves as an initial investigation into pinpointing the particular neural circuits affected by exposure to sevoflurane and provides early evidence suggesting that the activation of the mPFC‐amygdala neural circuit plays a role in the development of sevoflurane‐induced PND.

The manipulation of mPFC using chemogenetic techniques, whether to suppress or activate this area, was found to have little effect on the mice's movement abilities, as assessed by the open field test. Similarly, the inhibition of AMPA receptor function, mitochondrial stress, or the synthesis of glutamate in the amygdala did not result in any changes to locomotor activity. This suggests that the improvement in learning and memory deficits seen in mice exposed to sevoflurane, following specific interventions in the mPFC and amygdala, may not be linked to any alterations in movement. Additionally, the similar locomotor abilities displayed by both the Con group and the Sev group indicate that the learning and memory issues caused by sevoflurane are probably not a result of locomotor dysfunction.

In this research, we detected a minimal expression of the c‐Fos immediate early gene, a marker of neural activation, in the mPFC under standard conditions. However, sevoflurane exposure precipitated a pronounced elevation in c‐Fos‐positive cells within the mPFC. The temporal profile of this increase, persisting for 48 h after sevoflurane exposure, corresponds with the expected duration of sevoflurane‐related inflammatory processes [37, 38]. While prior studies demonstrate rapid c‐Fos activation by sevoflurane within minutes [39], our focus on the 4‐h timepoint aimed to evaluate sustained neuronal circuit activity linked to downstream neuropathological processes and cognitive deficits, which develop over hours post‐exposure. This rationale aligns with our hypothesis that prolonged circuit dysregulation—rather than acute activation—drives sevoflurane‐induced PND.

Sevoflurane exhibits concentration‐ and duration‐dependent duality, with low doses/short exposures conferring neuroprotection [40, 41] and prolonged/high‐dose exposure inducing neurotoxicity [42, 43]. Our study focused on the latter paradigm (4‐h, 3% sevoflurane) to model clinical scenarios of prolonged anesthesia in elderly patients at risk for PND. Our findings reflect the neurotoxic axis, where mPFC‐amygdala circuit overactivation drives downstream AMPAR‐mediated excitotoxicity and neuroinflammation. While preconditioning effects of sevoflurane are mechanistically distinct and often involve prosurvival signaling, our model does not preclude potential circuit‐specific adaptations at lower doses—a valuable avenue for future research. The rationale for selecting a single 4‐h sevoflurane exposure protocol is based on established preclinical models of PND in aged rodents, which aim to replicate prolonged anesthesia during major surgeries in elderly patients. Clinically, surgeries such as joint replacements or abdominal procedures in older adults often last 3–6 h, and our 4‐h exposure aligns with this timeframe to model sustained anesthesia. While human anesthesia practices vary in duration and drug combinations, this protocol isolates sevoflurane's effects under controlled conditions, enabling mechanistic insights into PND pathogenesis. However, our model provides a validated framework to study neurocognitive outcomes linked to prolonged anesthesia, as even single prolonged exposures in aged mice reliably induce PND‐like deficits [44].

The mPFC is considered essential in social engagement, encoding fear‐related memories, and memory and cognition processing of such recollections [45, 46]. Notably, there is a straightforward excitatory projection connecting the mPFC to the amygdala, and stimulation of the fasciculus retroflexus [47] has been shown to activate glutamatergic neurons within the amygdala [48]. In congruence with previous research, our findings propose the existence of neural projections from the mPFC to the amygdala, positing that the mPFC may exert regulatory control over amygdala activity through glutamatergic transmission. This postulation is supported by the co‐localization of the fluorescent tracer mCherry with VGLUT1, a marker for glutamatergic neurons. Furthermore, suppression of mPFC neuronal activity was correlated with a decrease in the activation state of AMPA receptors within the amygdala. Direct inhibition of the mPFC, as well as the retrograde suppression of mPFC neurons via their projections to the amygdala, mitigated sevoflurane‐induced cognitive deficits. Conversely, retrograde activation of mPFC neurons through their amygdala projections initiated cognitive deficits. These findings illuminate a potentially novel role for the mPFC–amygdala pathway in the etiology of sevoflurane‐induced cognitive deficits. This association may not be entirely unexpected given that the mPFC is implicated in the mediation of depression [12].

Our previous research has indicated that sevoflurane may lead to LTP suppression in mice exposed to sevoflurane [29, 36]. Our current investigation demonstrates that chemogenetic inhibition of mPFC imparts neuroprotection against sevoflurane‐induced neuronal apoptosis and LTP inhibition in the amygdala. These findings substantiate the role of the mPFC‐amygdala neural circuitry in the etiology of PND, contributing to the understanding of its underlying neuropathological mechanisms. Beyond influencing LTP, the attenuation of mPFC activation mitigated the sequelae of mitochondrial stress, inflammatory responses, and neuronal apoptosis within the amygdala of mice exposed to sevoflurane. These pathological processes are recognized contributors to cognitive deficits [49, 50]. Notably, neuroinflammation has been postulated as a primary neuropathological mechanism underlying PND [51]. Therefore, the mitochondrial stress, inflammatory processes, and neuronal apoptosis elucidated in the present study may represent fundamental neuropathological phenomena associated with PND.

AMPA receptors (AMPARs) as tetrameric ion channels predominantly enable excitatory neurotransmission in the central nervous system [52, 53]. These receptors are pivotal in facilitating synaptic plasticity, a cellular process essential for learning and memory [54, 55]. Activating AMPARs leads to the overload of intracellular calcium information and the release of calcium ions, which in turn activates cell death pathways, such as apoptosis and necrosis [56]. AMPA receptors are tetrameric structures composed of four subunits, designated as GluA1, GluA2, GluA3, and GluA4 [57]. Among these, the GluA1 subunit is particularly noteworthy for its role in controlling the calcium permeability and overall conductance of the receptor [58]. In this study, an increase in GluA1 expression in the amygdala was noted after sevoflurane exposure, supporting the hypothesis that sevoflurane activates the mPFC‐amygdala circuitry. Prolonged AMPAR activation may lead to calcium ion overaccumulation within cells, which in turn can initiate mitochondrial stress [59]. The investigations conducted in our study revealed that the administration of CNQX, an antagonist of AMPARs, directly into the amygdala diminished the manifestations of mitochondrial stress, the inflammatory response, and neuronal apoptosis within the amygdala and ameliorated the cognitive deficits observed in mice exposed to sevoflurane. These findings unequivocally implicate the activation of AMPARs in the amygdala as a contributory factor to the neuropathological alterations and the perturbations of learning and memory functions triggered by sevoflurane exposure.

Amygdala is known to have a high concentration of glutamatergic neurons [55, 60]. In our study, injection of glutamine antagonist BSO directly into the amygdala helped mitigate the learning and memory impairments caused by sevoflurane exposure. These results support the idea that the activity of glutamatergic neurons in the amygdala plays a crucial role in the underlying mechanisms of sevoflurane‐induced PND.

Mitochondria are crucial for various cellular activities, such as intercellular communication, ATP production, calcium homeostasis, and metabolic functions [61, 62, 63]. Mitochondria are highly responsive to both intracellular and extracellular perturbations. Disruptions such as calcium imbalances, hypoxic conditions, inflammatory processes, and oxidative stress can destabilize mitochondrial homeostasis, resulting in mitochondrial dysfunction [64, 65]. SOD2 is a critical antioxidant enzyme located in the mitochondria, where it functions to neutralize reactive oxygen species (ROS), particularly the superoxide anion [66, 67]. Under mitochondrial stress, such as during oxidative stress or metabolic dysfunction, ROS production is elevated, which can damage mitochondrial components and exacerbate cellular dysfunction [68]. In response, SOD2 levels are boosted to keep reactive oxygen species (ROS) in check and safeguard the mitochondria's proteins, fats, and DNA from oxidative damage. By regulating ROS levels, SOD2 ensures the health and functionality of the mitochondria, thus mitigating the adverse effects of mitochondrial stress [69]. In contrast, cytochrome c is a key player found in the inner mitochondrial membrane, and it is pivotal in the process of apoptosis. When the mitochondria are under stress, their membrane integrity gets compromised, causing cytochrome c to be released into the cytoplasm [70, 71]. Upon entering the cytosol, cytochrome c attaches to apoptotic protease activating factor 1 (Apaf‐1), activating caspase‐3, which starts the apoptotic cascade and results in cell death [72, 73]. As suggested by the present study, administration of the mitochondrial stress inhibitor NAC into the amygdala decreased the levels of SOD2 and cytochrome c in mice that were exposed to sevoflurane. NAC also diminished inflammation and apoptosis and led to cognitive improvements in mice exposed to sevoflurane. These results indicate a crucial involvement of mitochondrial stress in the development of PND.

In the present study, administration of the mitochondrial stress inhibitor NAC into the amygdala decreased the levels of SOD2 and cytochrome c in mice that were exposed to sevoflurane. NAC also diminished inflammatory responses and neuronal apoptosis and led to cognitive improvements in mice exposed to sevoflurane. These findings point toward a significant role of mitochondrial stress in the pathogenesis of PND.

Our data in the present study indicate that the activation of the mPFC‐amygdala neural circuit is both a requisite and contributory factor for the development of PND in mice exposed to sevoflurane. This phenomenon is mediated via the activation of glutamatergic neurons within the amygdala. Consequential to this activation is the induction of mitochondrial stress and an inflammatory response, culminating in a reduction of neuronal apoptosis and LTP suppression. These events delineate a potential mechanistic pathway underlying both the immediate and sustained cognitive impairments characteristic of PND [74, 75, 76].

The present study acknowledges several limitations. Notably, the LTP suppression and a decrement in glutamatergic neuronal populations within the amygdala were observed after sevoflurane exposure. Given the extensive glutamatergic innervation from the mPFC to the amygdala [77, 78], it might be postulated that these glutamatergic neurons are implicated in PND pathogenesis. This supposition gains credence from results demonstrating that BSO treatment ameliorated sevoflurane‐induced cognitive deficits. Nevertheless, it is imperative to acquire further evidence to definitively rule out the involvement of the amygdala's non‐glutamatergic neurons in sevoflurane‐induced cognitive deficits. Additionally, the study inferred AMPA receptor activity through phosphorylation levels of the GluA1 subunit. While not directly substantiated by electrophysiological assays, the observed reduction in GluA1 phosphorylation following CNQX administration does lend some support to the use of GluA1 phosphorylation as a surrogate marker for AMPA receptor activation. Lastly, the research was conducted exclusively on male mice, which presents a gender‐specific view of the PND pathology. Future research incorporating female mice would be essential to ascertain whether the findings reported herein exhibit sexual dimorphism.

## 5 Conclusions

**FIGURE 12:**
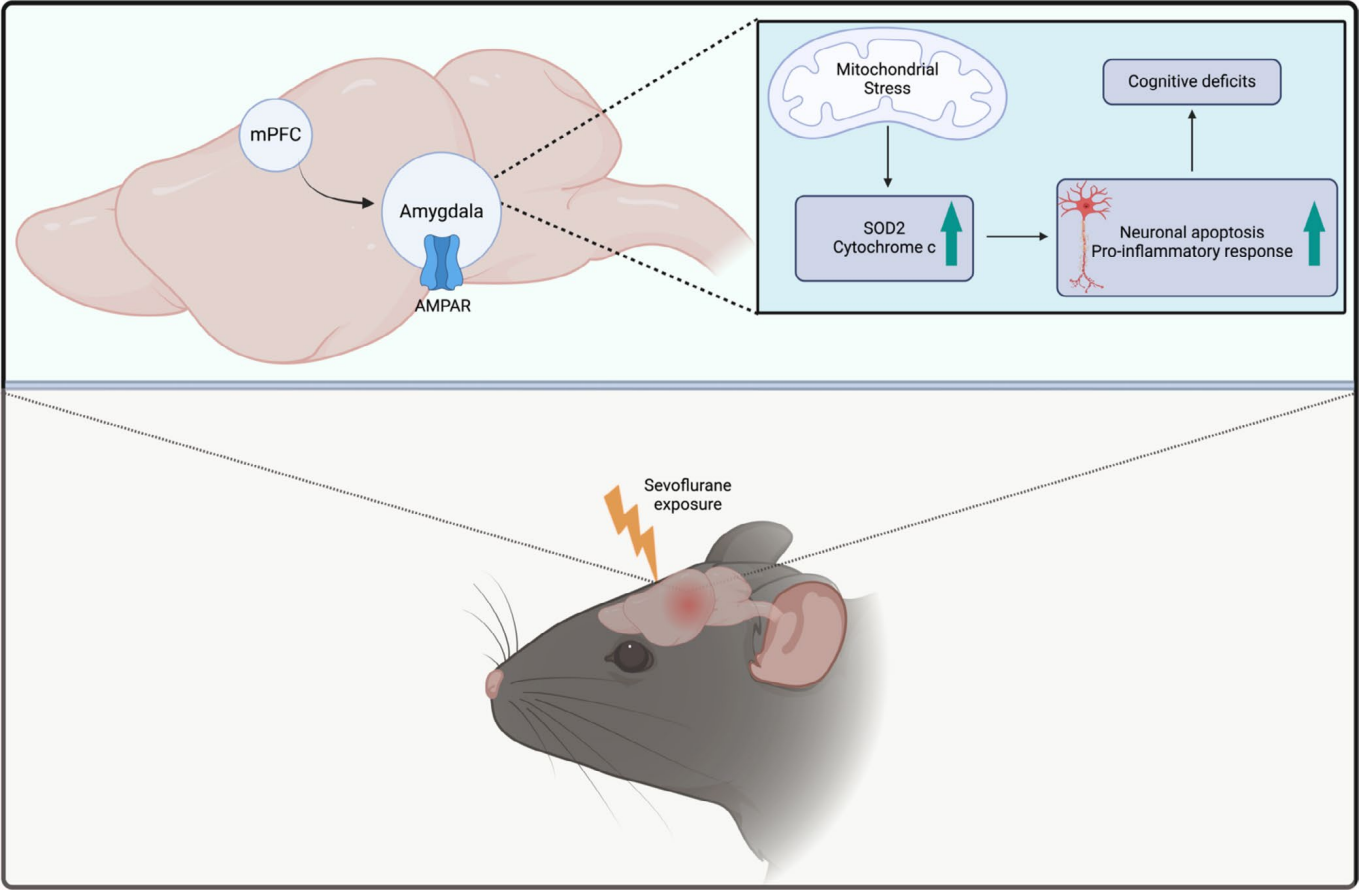
The schematic depicting potential neural circuits and molecular mechanisms involved in sevoflurane‐induced cognitive deficits. Sevoflurane exposure activates the mPFC neurons, which leads to mitochondrial stress, inflammatory responses, and neuronal apoptosis in the amygdala, leading to a subsequent LTP suppression observed in the amygdala.

In summary, the findings in the present study propose new insights into the neuronal and molecular underpinnings responsible for the onset of sevoflurane‐induced PND. Specifically, sevoflurane is postulated to initiate activation within mPFC neurons. This activation precipitates a cascade of cellular stress responses, evidenced by mitochondrial stress, subsequent inflammatory reactions, and neuronal apoptosis within the amygdala, leading to a subsequent LTP suppression observed in the amygdala (Figure 12).

## Author Contributions

Junhua Li: Conceptualization, methodology, investigation, and drafted the manuscript. Jinbei Wen and Meigu Zeng: Methodology, investigation, and data analysis. Jinghong Mei and Cong Zeng: Data curation. Ning Liufu and Yujuan Li: Supervision and revised the manuscript. All authors reviewed and endorsed the final manuscript.

## Conflicts of Interest

The authors declare no conflicts of interest.

## Supporting information


**Figure S1.** The mPFC’s projection fibers exhibit co‐localization with VGLUT1 but not with VGAT. (A) Representative immunofluorescent images showing the colocalization of mCherry with VGLUT1 in the amygdala. (B) Representative immunofluorescent images showing the colocalization of mCherry with VGAT in the amygdala. Scale bar: 20 μm.

**Figure S2.** Successful transduction was achieved in over 80% of mPFC neurons. (A, B) Representative immunofluorescent images (A) and quantification (B) of mice receiving injection of AAV‐hSyn‐hM4Di‐mCherry or AAV‐hSyn‐mCherry into the mPFC. Scale bar: 100 μm.

## Data Availability

The data supporting the findings of this study are available from the corresponding author upon reasonable request.
