# Supplementary figures and images for: Suppression of mPFC‐Amygdala Circuit Mitigates Sevoflurane‐Induced Cognitive Deficits in Aged Mice
Source: CNS Neurosci Ther. 2025 May 16;31(5):e70443. doi: 10.1111/cns.70443 (PMC12082282; doi:10.1111/cns.70443)

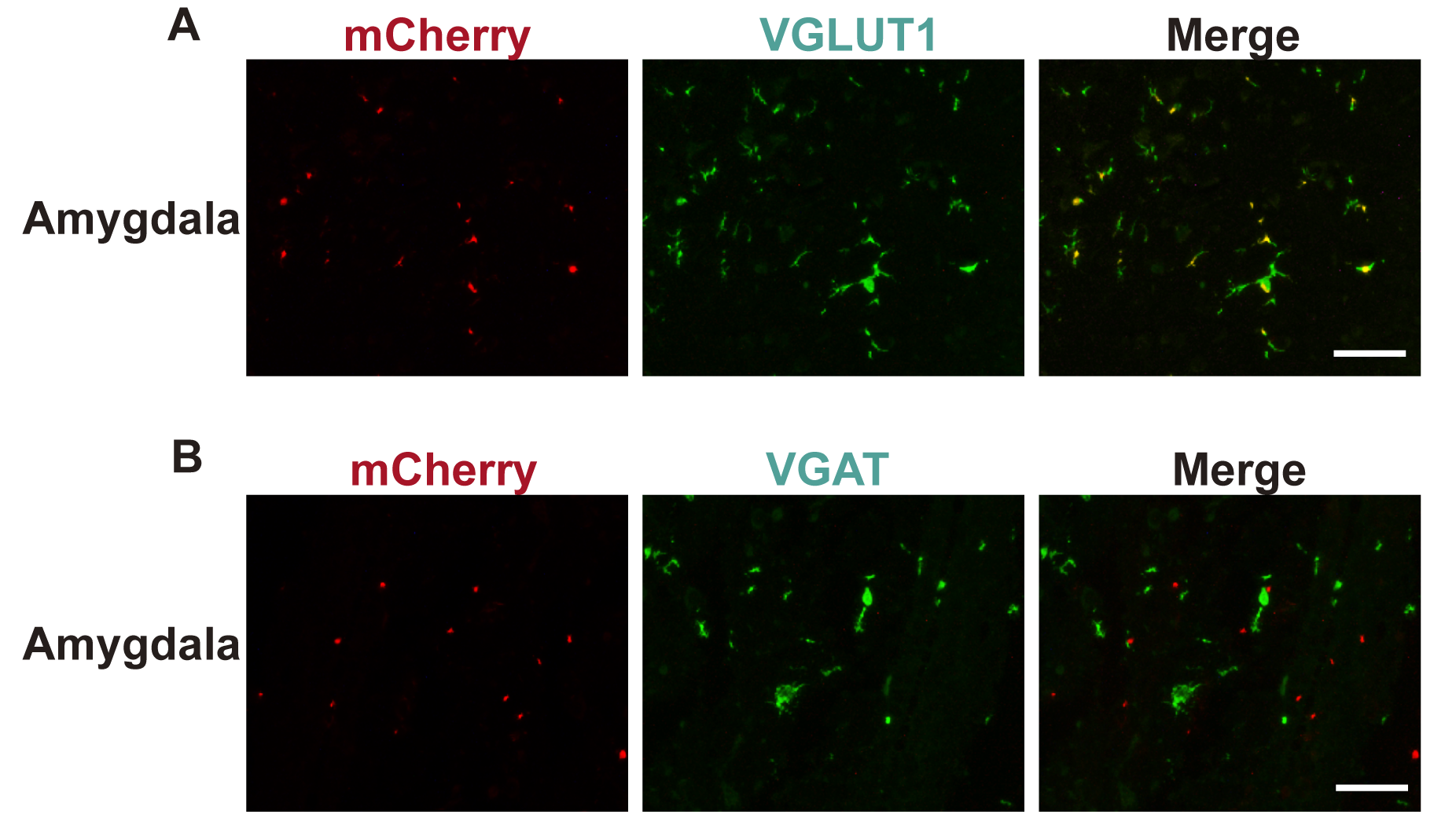

Supplement: Supplementary file 1 — Figure S1. The mPFC’s projection fibers exhibit co‐localization with VGLUT1 but not with VGAT. (A) Representative immunofluorescent images showing the colocalization of mCherry with VGLUT1 in the amygdala. (B) Representative immunofluorescent images showing the colocalization of mCherry with VGAT in the amygdala. Scale bar: 20 μm. [file CNS-31-e70443-s001.tif]

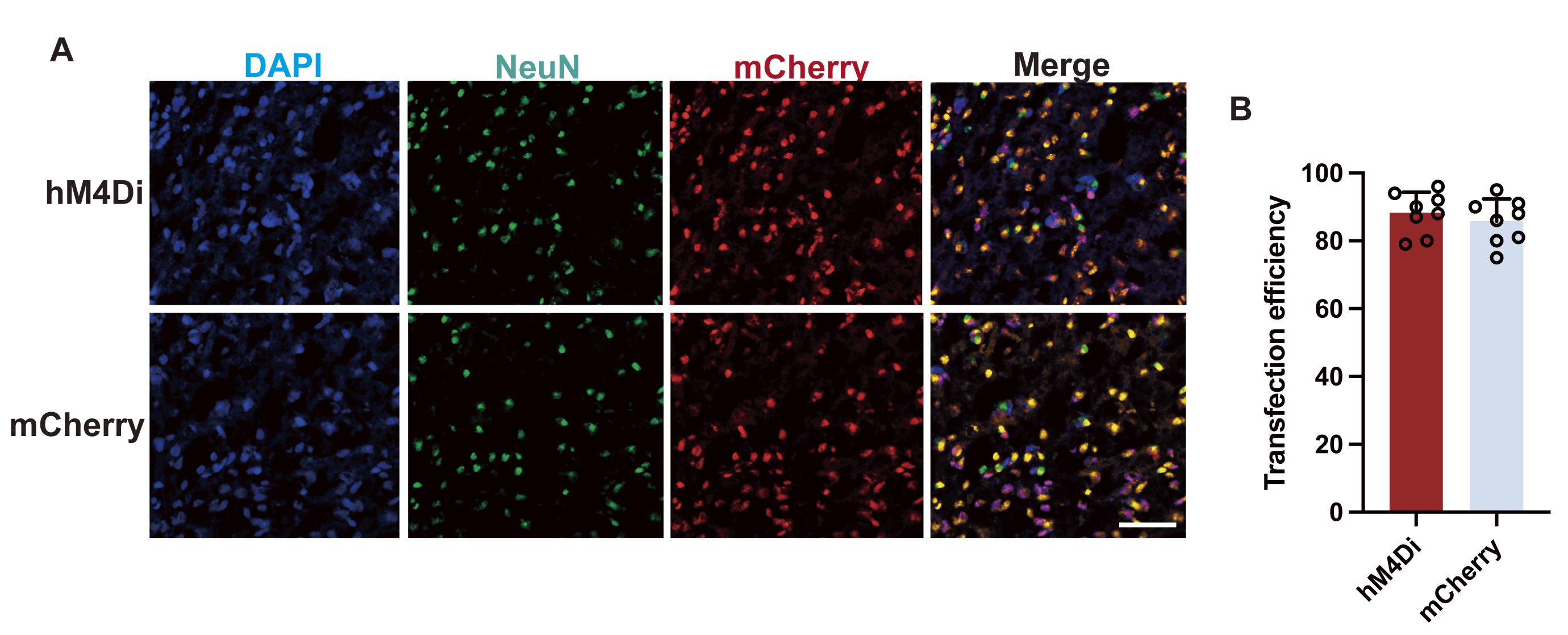

Supplement: Supplementary file 2 — Figure S2. Successful transduction was achieved in over 80% of mPFC neurons. (A, B) Representative immunofluorescent images (A) and quantification (B) of mice receiving injection of AAV‐hSyn‐hM4Di‐mCherry or AAV‐hSyn‐mCherry into the mPFC. Scale bar: 100 μm. [file CNS-31-e70443-s002.tif]
